# Identification and Validation of Reference Genes for Gene Expression Analysis in *Monochamus saltuarius* Under *Bursaphelenchus xylophilus* Treatment

**DOI:** 10.3389/fphys.2022.882792

**Published:** 2022-04-25

**Authors:** Jiaxing Li, Ningning Fu, Lili Ren, Youqing Luo

**Affiliations:** Beijing Key Laboratory for Forest Pest Control, Beijing Forestry University, Beijing, China

**Keywords:** *Monochamus saltuarius*, *Bursaphelenchus xylophilus*, RT-qPCR, reference genes, developmental stages

## Abstract

A special mutual relationship exists between the pine wood nematode (PWN) *Bursaphelenchus xylophilus* and its vector beetles of genus *Monochamus*, which enables PWN to spread, at the same time provides longhorned beetles with more weak hosts. PWN are attracted to the pupal chambers and then carried inside the trachea of beetle adults, which is a necessary part to complete the *B. xylophilus* infection cycle. The growth and immune responses of the vector beetle will affect this carrying process, however, they were rarely studied in *Monochamus saltuarius*. Real-time quantitative polymerase chain reaction (RT-qPCR), one of the most common methods for quantitative gene expression analysis, was performed to explore the key genes and pathways involved in the growth, development and immune responses of *M. saltuarius* at different developmental stages associated with infection of PWN and PWN treatment conditions. To enhance the accuracy of RT-qPCR data, the expression of target genes needs to be normalized with reference genes, which are stably expressed under varied experimental conditions. In our study, the stability of 14 candidate reference genes in *M. saltuarius* samples at different developmental stages associated with infection of PWN or PWN treatment conditions was evaluated using delta Ct, geNorm, NormFinder, BestKeeper and RefFinder algorithms. Moreover, *KLF* gene was used to validate the stability of the selected reference genes. Under experimental conditions of this study, *RPL7* and *TER* were suitable reference genes at different developmental stages associated with infection of PWN. *RPL7* and *RPS5* were considered the most stable reference genes in the pupae treated with PWN. *RPS5* and *SNX6* could be used as reference genes in the adults treated with PWN. *RPL7*, *EF1-γ*, and *RPS5* could be used as stable reference genes in all the samples. This work is the first to evaluate reference genes in *M. saltuarius*, laying a foundation for further gene expression experimental procedures and understanding the phoretic relationship between *M. saltuarius* and *B. xylophilus*.

## Background

Pine wilt disease is one of the most dangerous and devastating diseases caused by *Bursaphelenchus xylophilus* (pine wood nematode; PWN) worldwide. *B. xylophilus* originated in North America, and was introduced to Japan, Korea, China, Portugal, and other countries, causing serious damage in these invasion areas (Dropkin and Foudin, 1979; Mamiya, 1988; Cheng et al., 1983; Han et al., 2008; Khan, 1991; Manuel et al., 1999; Abelleira et al., 2011). PWN is transmitted to dead or dying trees by its insect vector, the *Monochamus* beetles, during oviposition or maturation feeding (Akbulut and Stamps, 2012; Kim et al., 2020; Li et al., 2020).

In China, PWN was first widely spread and damaged in southern area, then spread to northern and other regions. In the southern area, *Monochamus alternatus* as a main vector of the PWN was widely studied. However, *Monochamus saltuarius* emerged as a new and unique vector in Liaoning Province, China (Yu and Wu, 2018), although it was regarded as a common vector in Korea and Japan (Sato, 1987; Kim et al., 2006). It greatly promotes the transmission of *B. xylophilus* to *Larix* spp., *Pinus koraiensis*, *Pinus sylvestris* var. *mongolica*, and *Pinus tabuliformis* (Yu and Wu, 2018; Yu et al., 2019; 2020). Therefore, the research about interaction between *B. xylophilus* and *M. saltuarius* is of significance to prevent and control the prevalence of pine wilt disease in north of China.

Gene expression is an important method to study the potential function of insect genes in different conditions. Real-time quantitative polymerase chain reaction (RT-qPCR), one of the most common methods for quantitative gene expression analysis, has the characteristics of high accuracy, specificity, sensitivity, and rapidity (Bustin, 2002; Bustin et al., 2005; Valasek and Repa, 2005). However, the quality and quantity of RNA extraction, polymerase amplification efficiency, and cDNA synthesis efficiency can all lead to systematic errors during RT-qPCR operation (Klein, 2002; Fleige and Pfaffl, 2006). To eliminate these errors, various strategies have been used to normalize RT-qPCR data, and using internal controls or reference genes has become the most reliable method (Silver et al., 2006; Borowski et al., 2014; Silveira et al., 2021). Nevertheless, there are no absolute stable reference genes because of spatio-temporal specificity of genes and variable experimental conditions. So, it is necessary to screen suitable reference genes according to specific experimental materials and conditions for RT-qPCR analysis.

Studies of reference genes in insects are common. In coleoptera, reference gene screening has been performed in approximately twenty insect species, such as *Dendroctonus valens*, *Harmonia axyridis*, and Cerambycidae species*, Monochamus alternatus, Anoplophora glabripennis* (Toutges et al., 2010; Rajarapu et al., 2012; Shi et al., 2013; Barros Rodrigues et al., 2014; Wang et al., 2014; Song et al., 2015; Tan et al., 2015; Feng et al., 2016; Rodrigues et al., 2017; Yang et al., 2018; Yang et al., 2020; Zhang et al., 2020; Zheng et al., 2020; Guo et al., 2021; Sellamuthu et al., 2021). And common reference genes for Coleoptera studies include *ACT* (actin), *β-TUB* (beta-tubulin), *α-TUB* (alpha-tubulin), *RPs* (ribosomal proteins), *18S rRNA* (18S ribosomal RNA), *28S* rRNA (28S ribosomal RNA), *EF1-α* (elongation factor1-α) and so forth (Toutges et al., 2010; Zhang et al., 2020; Guo et al., 2021). These genes are involved in normal cell metabolic processes. However, reference genes in *M. saltuarius* have not been reported. Therefore, we required to find the appropriate reference genes for gene expression analysis under different PWN treatments.

In this study, we aimed to identify the optimal reference genes in *M. saltuarius* at different developmental stages associated with infection of PWN or PWN treatment conditions. Based on prior experimental reports regarding reference genes in Coleoptera and other insects, 14 candidate reference genes including sorting nexin 6 (*SNX6*), phospholipid-transporting ATPase (*ATPase*), palmitoyltransferase ZDHHC15 isoform X2 (*ZDhhc15*), transcription factor A, mitochondrial-like (*TFAM*), 60S ribosomal protein L18 (*RPL18*), 60S ribosomal protein L7 (*RPL7*), 40S ribosomal protein S5 (*RPS5*), transitional endoplasmic reticulum ATPase TER94 (*TER*), transmembrane and ubiquitin-like domain-containing protein 1 (*Tmub1*), eukaryotic translation initiation factor 4B (*EIF*), elongation factor 1-gamma (*EF1-γ*), cytochrome c oxidase subunit 7C (*COX7*), tubulin alpha-1 chain (*α-TUB*), and triosephosphate isomerase (*TPI*) were selected from the genome and transcriptome data of *M. saltuarius* (unpublished data). Five algorithms were used to evaluate reference genes stability and perform a comprehensive ranking. In addition, the expression profile of the Krüppel-like factor luna (*KLF*) gene was used to verify our result. This study provides valuable information for further exploration on the growth and immune mechanism of *M. saltuarius*, and serves as a reference for exploring its phoretic relationship with *B. xylophilus*.

## Materials and Methods

### Insect

In August and December 2020, the fourth and fifth instar larvae of *M. saltuarius* were collected from Dahuofang Forest Farm, Fushun City, Liaoning Province, China. To ensure the absence of *B. xylophilus* all times, after sterilizing the larvae surface with 75% alcohol, the fifth instar larvae were incubated at artificial media at 25°C with 75% relative humidity. All procedures were performed at the Plant Quarantine Laboratory, Beijing Forestry University, Beijing, China.

### Sample Treatment

Samples collected at different developmental stages associated with infection of PWN in *M. saltuarius* included two instar larval stages (L4 and L5), 1-day-age pupae (P1), 5-days-age pupae (P5), 10-days-age pupae (P10), and newly emerged adult (1-day-old) males (AM) and females (AF). Three independent biological replicates were performed at each stage, and each replicate was derived using an individual beetle. All samples were immediately frozen in liquid nitrogen and stored at −80°C for RNA extraction.

To test the effect of *B. xylophilus* on *M. saltuarius*, the artificial co-culture medium of *B. xylophilus* and *M. saltuarius* was prepared using the previous method (Li et al., 2021). Fifth instar larvae with weights ranging from 300 to 500 mg were selected from Dahuofang Forest Farm in December 2020. After sterilizing the larvae surface with 75% alcohol, the fifth instar larvae inoculated in the artificial co-culture medium were cultured with *B. xylophilus* at 25°C and 75% relative humidity. The growth and developmental stage of beetles was observed once every 24 h. After the beetle larvae pupated, 5-days-age pupae (BP5), 10-days-age pupae (BP10), and newly emerged adult (1-day-old) males (BAM) and females (BAF) were collected respectively. Three biological replicates were performed at each stage, and each replicate included one sample. All samples were immediately frozen in liquid nitrogen and stored at −80°C for RNA extraction.

### RNA Extraction and cDNA Synthesis

Total RNA from all 33 samples at different developmental stages associated with infection of PWN and PWN treatment conditions were extracted using EASY Spin Plus Tissue/Cell RNA Extraction Kit (Aidlab, China). Quality and quantity of total RNA were evaluated using 1.2% (w/v) agarose gel electrophoresis and NanoDrop 2,000 spectrophotometry. The PrimeScript^™^ RT Reagent Kit (Takara, China) was used to synthesize the first strand cDNA of each sample according to the manufacturer’s protocol. Obtained cDNAs were diluted 5-fold and stored at −20°C for subsequent RT-qPCR experiments.

### Selection of Candidate Reference Genes and Primer Design

Fourteen candidate reference genes with relatively high transcript abundance and stable expression [fragments per kilobase of transcript per million mapped reads (FPKM) value >20 and a fold change in expression <2] were selected based on the transcriptome and genome data (unpublished data) of *M. saltuarius*. These genes were *SNX6*, *ATPase*, *ZDhhc15*, *TFAM*, *RPL18*, *RPL7*, *RPS5*, *TER*, *Tmub1*, *EIF*, *EF1-γ*, *COX7*, *α-TUB*, and *TPI* (Supplementary Table S1). Their coding DNA sequences were obtained from *M. saltuarius* genome data (GeneBank: OM471799–OM471813), and primers were designed using the web software Primer 3.0 (https://bioinfo.ut.ee/primer3-0.4.0/) and IDT (https://sg.idtdna.com/pages). The primers used for amplification are listed in Table 1.

**TABLE 1:** Primer sequences and amplification characteristics of candidate reference genes.

Accession Number	Symbol	Gene Name	Primer sequence (5′to3′)	Size (bp)	E (%)	*R* ^2^ Value
Gene_ MSAL09320	*SNX6*	sorting nexin-6	F: CGT​TAT​GAG​GAG​GAA​CCC​AAA​TA	119	97	0.998
			R: CTC​ATG​GTT​CCT​TCA​CCT​TCT​C			
Gene_ MSAL04397	*ATPase*	probable phospholipid-transporting ATPase	F: GAA​CTC​GGC​AGG​ATC​TCT​TAT​T	99	90	0.994
			R: ATA​GCT​GAC​CGT​ACC​CAA​ATG			
Gene_ MSAL02314	*ZDhhc15*	Palmitoyltransferase ZDHHC15 isoform X2	F: CGA​GGT​GTT​GGT​ACA​GAC​AAA	144	109	0.998
			R: GCG​TGA​GTG​TAC​AGG​GTA​TTC			
Gene_ MSAL10600	*TFAM*	transcription factor A, mitochondrial-like	F: CAA​TGG​CAG​ACT​GGG​AAG​AA	115	97.1	0.991
			R: CTG​CCT​GGT​TTC​AAC​TGT​CTA			
Gene_ MSAL00760	*RPL18*	60S ribosomal protein L18	F: AAC​GGT​ATT​GAT​GCA​AGG​TAG​A	103	104.2	0.998
			R: GGA​ACG​TAC​TAG​TGG​CTT​AGT​G			
Gene_ MSAL00096	*RPL7*	60S ribosomal protein L7	F: GGC​AAC​GCA​TTC​CCA​TAA​C	109	105.4	0.998
			R: CTT​GGA​CCG​ACT​GTG​AAG​AT			
Gene_ MSAL00148	*RPS5*	40S ribosomal protein S5	F: CGT​AGG​GTA​AAC​CAG​GCT​ATC	122	94.7	0.999
			R: GAG​GAA​CCC​TTA​GCA​GCA​TTA			
Gene_ MSAL03702	*TER*	transitional endoplasmic reticulum ATPase TER94	F: GTC​GTT​GCT​CTT​TCA​CAA​GC	203	100.2	0.998
			R: CAA​GGC​TGG​ATG​GAC​ACT​AC			
Gene_ MSAL09667	*Tmub1*	transmembrane and ubiquitin-like domain-containing protein 1	F: CGT​AGT​CTG​CCT​TCT​GAC​AAT​AA	97	90.9	0.999
			R: ACA​TCT​CCT​CCA​ACC​TAC​CA			
Gene_ MSAL09352	*EIF*	eukaryotic translation initiation factor 4B	F: CGA​CGA​TAG​GGA​TGA​TCG​TAA​AG	124	94.3	0.998
			R: CCT​TTC​CCT​TGG​TTC​TGA​CAT​A			
Gene_ MSAL06575	*EF1-γ*	elongation factor 1-gamma	F: ACA​GCA​ACG​CTA​TCG​CTT​AT	103	90.7	0.999
			R: TCA​CCT​TCG​GCA​AAT​CCT​ATC			
Gene_ MSAL03188	*COX7*	cytochrome c oxidase subunit 7C, mitochondrial-like	F: GTG​GTG​TAC​CTG​GAG​CGA​AT	114	91.8	1.000
			R: GTC​TCA​AGA​TGA​GGA​AAG​GTG​C			
Gene_ MSAL09430	*α-TUB*	tubulin alpha-1 chain	F: CCC​TTA​CCC​ACG​TAT​TCA​CTT​C	98	91.7	1.000
			R: TGG​TAA​TTT​CAG​CCA​CGG​ATA​G			
Gene_ MSAL06278	*TPI*	triosephosphate isomerase	F: ATC​GGT​GAG​ACC​TTA​GAG​GAA	102	94	0.999
			R: CAC​GTT​CGA​CCA​GTC​TTT​GA			
Gene_ MSAL01719	*KLF*	Krüppel-like factor luna	F: GCA​GAG​ACT​TTG​ACT​CCT​CCC	144	95.4	0.998
			R: GGC​TCG​CAC​TCT​GAC​TAT​TGT			

### RT-qPCR Analysis

RT-qPCR was performed using Bio-Rad CFX Connect real-time PCR instrument (Bio-Rad, United States) with TB Green^®^ Premix Ex Taq™ II (Takara, Japan). A 25-μl reaction volume consisted of 12.5 μl of TB Green Premix Ex Taq Ⅱ (2×), 1 μl of forward and reverse primers (10 μM), respectively, 1 μl of cDNA template, and 9.5 μl of RNA-free water. Amplification conditions were as follows: initial denaturation at 95°C for 30 s, followed by 40 cycles at 95°C for 5 s and 60°C for 30 s. Then, we performed a melt curve analysis using the default parameters with a steady increase in temperature from 65 to 95°C. All RT-qPCR assays were performed in three biological replicates, each biological replicate with three technical replicates. The amplification efficiency (E) and correlation coefficients (*R* ^2^) were determined for each gene using the standard curves with a 5-fold dilution series of the template (1, 1/5, 1/25, 1/125, and 1/625), where *R* ^2^ was the slope of the standard curve. Amplification efficiency was calculated according to the equation: E% = (10 [−1/slope]−1) × 100%.

### Stability Analysis of Candidate Reference Genes

Five algorithms, including delta Ct, geNorm, NormFinder, BestKeeper and RefFinder, were used to analyze the expression stability of the candidate reference genes in different groups. The delta Ct algorithm (based on the delta Ct method) was used to calculate the mean standard deviation (SD) of the paired genes in each sample to assess the gene expression stability. Genes with lower SD value had more stable expression (Silver et al., 2006). The GeNorm was used to calculate the M value based on the pairwise variation between two reference genes. If the M value was less than 1.5, it could be considered a suitable reference gene. The smaller the M value, the higher the stability of gene. The optimal number of reference genes was determined by calculating pairwise variation (V_n_/V_n+1_) by geNorm. A value of V_n_/V_n+1_ less than 0.15 indicated that the most suitable reference gene number is n without introducing n + 1 (Vandesompele et al., 2002). In NormFinder, the S value of reference gene according to variance analysis decided the stability of candidate reference genes. The lower the S value, the more stable they were (Andersen et al., 2004). Before using geNorm or NormFinder analysis, the original Ct values were converted to 2^−ΔCt^ values (ΔCt = original Ct value − lowest Ct value in each group). BestKeeper evaluated the expression stability of all candidate reference genes by calculating the SD and stability value (SV) based on the original Ct values. A gene could not be used as an internal reference gene if the SD value was more than 1 (Pfaffl et al., 2004). Similarly, genes with lower SD and SV values had more stable expression. Finally, the comprehensive ranking of candidate reference genes under different conditions was obtained according to RefFinder (Xie et al., 2012).

### Validation of Reference Genes

Krüppel-like transcription factor luna (*KLF*) belongs to a family of 15 different zinc finger proteins of the C2H2 type that are involved invertebrate development, and which controls cell proliferation, growth and differentiation. De Graeve et al. (2003) proposed that *KLF* was a novel transcriptional determinant of *Drosophila* development (De Graeve et al., 2003). Therefore, the *KLF* gene was selected as target gene to validate the stability of the selected reference genes based on the transcriptome data. The primers used are shown in Table 1.

We used RT-qPCR (method same as above) to detect the *KLF* expression level in *M. saltuarius* samples at different developmental stages associated with infection of PWN and PWN treatment conditions. The relative quantification of the *KLF* gene was calculated using the 2^−ΔΔCt^ method (Livak and Schmittgen, 2001). One-way analysis of variance (ANOVA) followed by post-hoc Tukey’s honestly significant difference (HSD) test on SPSS Statistics Software was used to determine the significance of *KLF* expression levels at different developmental stages associated with infection of PWN and PWN treatment conditions (Wu et al., 2021; Fu et al., 2022).

## Results

### Primer Performance Analysis of Candidate Reference Genes

A total of 14 candidate reference genes were selected for gene-normalization studies in different samples. RT-qPCR products showed a single peak in the melting curve analysis (Supplementary Figure S1) and 1.2% agarose gel electrophoresis showed a specific band for each gene (Supplementary Figure S2). The amplification efficiency (E) values of all candidate genes ranged from 90% (*ATPase*) to 109% (*ZDhhc15*), and regression analysis of all primer pairs showed a correlation coefficient (*R* ^2^) greater than 0.99 (Table 1). These results indicated that all primer pairs designed for the candidate reference genes had good efficiency and specificity in RT-qPCR amplification. Therefore, the primers of these candidate reference genes were used for further analysis.

### Expression Analysis of Selected Reference Genes

**FIGURE 1:**
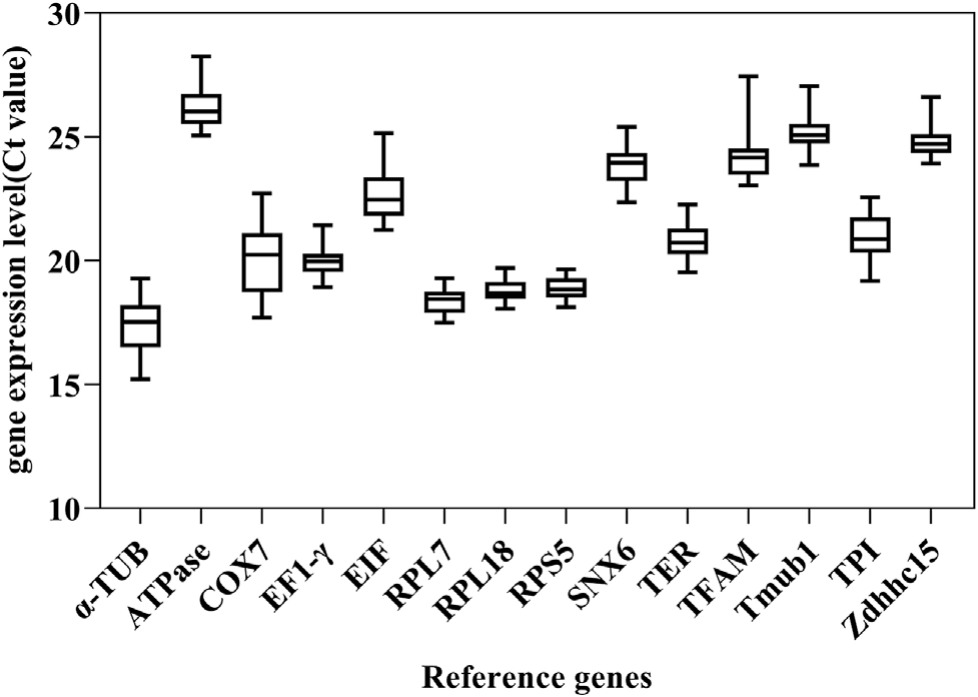
Cycle threshold (Ct) values of 14 candidate reference genes across total samples in *M. saltuarius*. Boxes indicate the 25th and 75th percentiles, and lines in the boxes represent the median value.

Transcript abundance and cycle threshold (Ct) variation are important parameters for screening reference genes. The Ct value of 14 candidate reference genes across 33 samples showed a wide range of expression levels and significant differences. Ct values of these candidate reference genes ranged from 14.98 to 27.14 for total samples. Among these, *α-TUB, RPL7* and *RPL18* were the most abundant transcripts (average Ct = 17.41, 18.34, 18.74, respectively). The least frequently expressed reference gene were *Zdhhc15*, Tmub1, and *ATPase* (average Ct = 24.80, 25.19, 26.21, respectively). According to the SD values, variance in Ct values increased in the following order: *RPS5 < RPL18 < RPL7 < EF1-γ < Zdhhc15 < TER < Tmub1 < SNX6 < ATPase < TPI < TFAM < EIF < α-TUB < COX7* (Figure 1).

### Expression Stability of Candidate Reference Genes in Different Developmental Stages Associated With Infection of PWN and PWN Treatments

To identify the most suitable reference genes of *M. saltuarius* under the three conditions—different developmental stages associated with infection of PWN, PWN treatment at the pupal stage, and PWN treatment at the adult stage, their expression stability was evaluated using five algorithms as elaborated below.

#### Delta Ct Analysis

For candidate reference genes in total samples, *RPL7*, *EF1-γ,* and *TER* were more stable than other reference genes (average SD = 0.73, 0.74, 0.76, respectively). For different treatments (Table 2), *TER*, *Zdhhc15* and *EF1-γ* had the most stable expression levels (average SD = 0.71, 0.73, 0.74, respectively) at different developmental stages associated with infection of PWN. *RPL7*, *RPS5*, and *TFAM* were the most stable reference genes (average SD = 0.57, 0.60, 0.65, respectively) in PWN-treated pupal groups; while *SNX6*, *RPS5*, and *RPL7* were the most stable reference genes (average SD = 0.40, 0.41, 0.43, respectively) in PWN-treated adult groups. On the whole *α-TUB*, *COX7*, and *TPI* were the least stable under most conditions.

**TABLE 2:** Expression stability ranking of the 14 candidate reference genes based on five algorithms.

Conditions	Genes	Delta Ct		geNorm		NormFinder		BestKeeper		RefFinder	
		Avg. Ct	Rank	M	Rank	SV	Rank	SD + CV	Rank	GM	Rank
	TER	0.71	1	0.55	6	0.27	1	0.54	6	2.45	1
	RPL7	0.76	4	0.38	1	0.36	6	0.44	3	2.91	2
	RPS5	0.89	9	0.38	2	0.43	9	0.40	1	3.08	3
	Zdhhc15	0.73	2	0.48	4	0.31	3	0.52	5	3.31	4
	EF1-γ	0.74	3	0.52	5	0.29	2	0.48	4	3.31	5
	RPL18	0.77	6	0.41	3	0.34	5	0.43	2	3.66	6
Developmental	Tmub1	0.77	5	0.56	7	0.33	4	0.58	7	5.60	7
Stages	SNX6	0.80	7	0.59	8	0.40	7	0.63	8	7.48	8
	ATPase	0.86	8	0.61	9	0.43	10	0.83	10	8.71	9
	TFAM	0.92	10	0.67	10	0.43	8	0.82	9	9.49	10
	α-TUB	1.00	12	0.71	11	0.57	13	0.86	12	11.72	11
	EIF	0.95	11	0.76	12	0.54	12	0.98	13	11.72	12
	TPI	1.01	13	0.80	13	0.49	11	0.86	11	12.49	13
	COX7	1.36	14	0.88	14	0.78	14	1.23	14	14.00	14
	RPL7	0.57	1	0.13	1	0.11	1	0.18	1	1.00	1
	RPS5	0.60	2	0.13	2	0.16	3	0.21	2	1.68	2
	RPL18	0.66	5	0.28	3	0.15	2	0.24	3	3.87	3
	EF1-γ	0.65	4	0.50	7	0.18	6	0.37	5	4.53	4
	TFAM	0.65	3	0.48	6	0.21	7	0.45	6	4.56	5
	Tmub1	0.75	7	0.36	4	0.17	4	0.35	4	5.29	6
Pupae treated	TER	0.70	6	0.43	5	0.18	5	0.46	7	5.96	7
with PWN	TPI	0.79	8	0.60	10	0.27	10	0.54	10	8.94	8
	ATPase	0.79	9	0.53	8	0.28	11	0.49	9	8.97	9
	SNX6	0.80	10	0.56	9	0.26	9	0.61	12	10.44	10
	EIF	0.80	11	0.63	11	0.21	8	0.60	11	10.46	11
	Zdhhc15	1.00	13	0.72	13	0.34	13	0.47	8	11.51	12
	α-TUB	0.96	12	0.67	12	0.30	12	0.82	14	12.47	13
	COX7	1.11	14	0.77	14	0.35	14	0.73	13	13.74	14
	RPS5	0.41	2	0.12	2	0.10	2	0.19	1	1.41	1
	SNX6	0.40	1	0.17	3	0.05	1	0.21	2	1.57	2
	RPL7	0.43	3	0.12	1	0.12	3	0.23	3	2.28	3
	EF1-γ	0.44	4	0.22	4	0.12	4	0.31	5	4.23	4
	Zdhhc15	0.48	5	0.27	5	0.20	8	0.29	4	4.73	5
	TFAM	0.50	6	0.30	6	0.13	5	0.40	9	6.64	6
Adults treated	Tmub1	0.52	7	0.34	7	0.18	6	0.42	10	7.65	7
with PWN	TER	0.56	8	0.37	8	0.23	12	0.47	11	8.66	8
	COX7	0.59	11	0.45	11	0.23	10	0.34	6	9.45	9
	ATPase	0.58	9	0.40	9	0.19	7	0.55	12	9.67	10
	TPI	0.60	12	0.47	12	0.23	11	0.38	7	10.49	11
	EIF	0.59	10	0.42	10	0.22	9	0.63	13	10.68	12
	RPL18	0.64	13	0.49	13	0.25	13	0.39	8	11.51	13
	α-TUB	0.79	14	0.54	14	0.30	14	0.73	14	14.00	14
	RPL7	0.73	1	0.32	1	0.26	2	0.43	3	1.57	1
	EF1-γ	0.74	2	0.48	4	0.23	1	0.46	4	2.38	2
	RPS5	0.83	6	0.32	1	0.39	7	0.39	1	2.55	3
	RPL18	0.81	5	0.44	3	0.33	5	0.39	2	3.50	4
	TER	0.76	3	0.56	6	0.30	3	0.57	7	4.41	5
	Tmub1	0.77	4	0.53	5	0.31	4	0.56	6	4.68	6
Total	Zdhhc15	0.85	8	0.61	8	0.38	6	0.50	5	6.62	7
samples	SNX6	0.83	7	0.59	7	0.39	8	0.64	8	7.48	8
	ATPase	0.85	9	0.63	9	0.41	9	0.77	10	9.24	9
	TFAM	0.91	10	0.67	10	0.43	10	0.72	9	9.74	10
	EIF	0.94	11	0.72	11	0.46	11	0.84	12	11.24	11
	TPI	1.08	12	0.81	13	0.60	12	0.80	11	11.98	12
	α-TUB	1.09	13	0.76	12	0.63	13	0.95	13	12.74	13
	COX7	1.46	14	0.90	14	0.95	14	1.31	14	14.00	14

Note: Avg. Ct, average cycle threshold; M, expression stability value; SV, stability value; SD + CV, standard deviation and coefficient of variation; GM, geometric mean.

#### GeNorm Analysis

**FIGURE 2:**
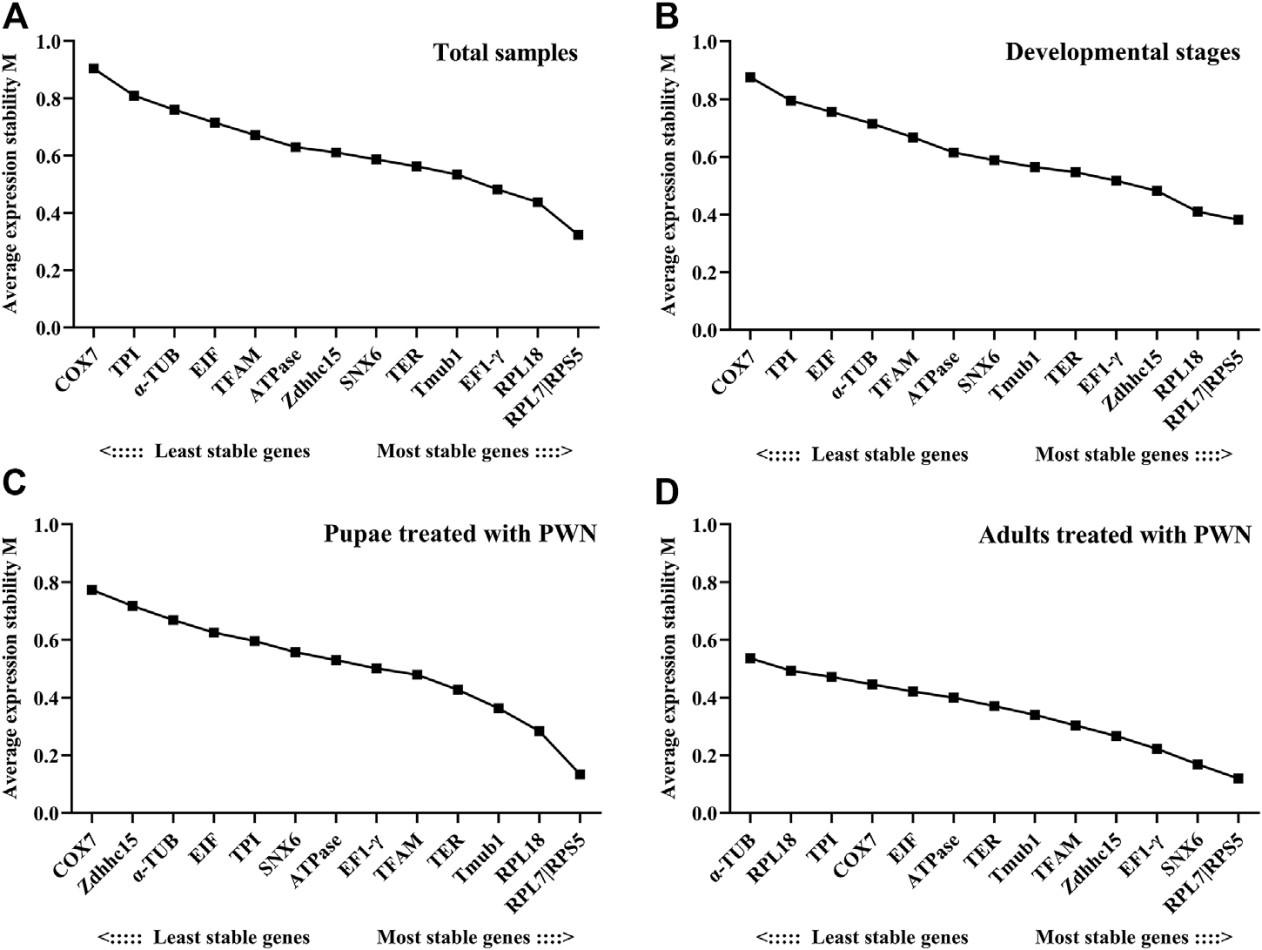
Average expression stability and ranking of candidate reference genes calculated by geNorm. Candidate reference genes with lower M values were more stable. The least stable genes are listed on the left, and the most stable genes are listed on the right. **(A)** Total samples. **(B)** Developmental stages associated with infection of PWN. **(C)** Pupae treated with PWN. **(D)** Adults treated with PWN.

Among all samples, *RPL7* and *RPS5* were the most stable reference genes, similar with the results found in the sample sets of different developmental stages associated with infection of PWN and all PWN treatment conditions (Figure 2). In addition, we found that the most unstable genes greatly varied in different experimental conditions. In developmental stages associated with infection of PWN and total samples, the M values of *COX7* and *TPI* were higher than other genes. In pupae treated with PWN, *COX7* and *Zdhhc15* had the least stability. In adults treated with PWN, *α-TUB* and *RPL18* exhibited the most unstable expression levels.

**FIGURE 3:**
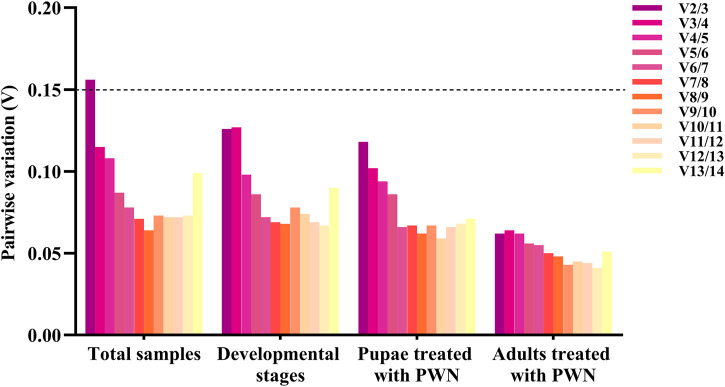
Pairwise variation (V) of 14 reference genes in different conditions calculated by geNorm. The threshold value for assessing the optimal number of reference genes for RT-qPCR normalization is 0.15.

The pairwise changes (V_n_/V_n+1_) were calculated using geNorm with a threshold value of 0.15 to assess the number of reference genes for all treatment conditions. Three groups including different developmental stages associated with infection of PWN, pupae treated with PWN, and adults treated with PWN, V2/3 values were all <0.15 (0.126, 0.118, and 0.062, respectively), indicating that two reference genes were sufficient for RT-qPCR normalization. In 33 total samples, the V2/3 value was 0.156, which was greater than the split-off value, and the V3/4 value was 0.115. Therefore, three reference genes were needed to normalize the expression of the target gene in all samples (Figure 3).

#### NormFinder Analysis

At different developmental stages associated with infection of PWN, the most stable genes were *EF1-γ* and *TER*. In pupae treated with PWN, *RPL7*, and *RPL18* had the strongest stability (Table 2). In adults treated with PWN, *SNX6*, and *RPS5* were the most stable reference genes. In total samples, *EF1-γ* and *RPL7* were the best reference genes combination. Unsurprisingly, *α-TUB* and *COX7* were also the least stable genes in most cases.

#### BestKeeper Analysis

**FIGURE 4:**
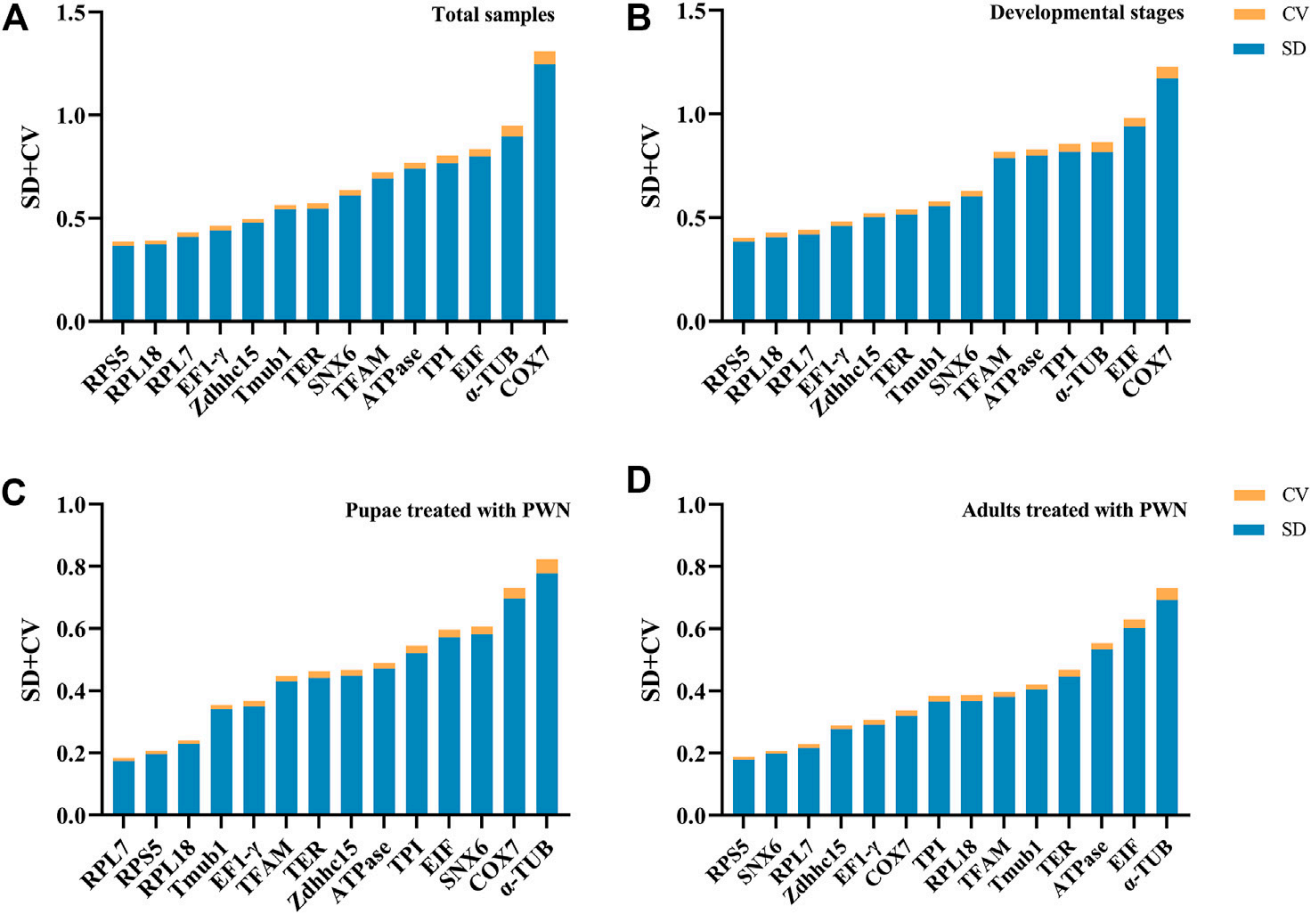
Stability rankings of 14 candidate reference genes by BestKeeper. Blue bars represent standard deviation (SD) of average Ct values, and yellow bars represent coefficients of variation (CV). **(A)** Total samples. **(B)** Developmental stages associated with infection of PWN. **(C)** Pupae treated with PWN. **(D)** Adults treated with PWN.

For BestKeeper algorithm, the most stable genes showed the lowest SD ± CV values, and genes with an SD value >1 were considered unstable. In total samples, *RPS5*, *RPL18,* and *RPL7* were the most stable genes. For different developmental stages associated with infection of PWN and pupae treated with PWN, two conditions had similar results that *RPS5*, *RPL18*, and *RPL7* were identified as the most stable genes, but *α-TUB*, *COX7*, *EIF*, and *SNX6* were poor stable genes. Whereas the PWN-treated adult group was slightly different from the above. For adults treated with PWN, *RPS5*, *SNX6*, and *RPL7* showed the highest stability; *ATPase*, *α-TUB*, and *EIF* had the least stable expression level (Figure 4). In some groups, *COX7*, whose SD value was greater than 1, was considered an unstable reference gene.

#### RefFinder Analysis: Comprehensive Stability Analysis of Reference Genes

The RefFinder program was used to obtain a comprehensive reference gene ranking based on the geometric mean of four algorithms. The expression stabilities of candidate reference genes in all samples decreased in the order: *RPL7 > EF1-γ > RPS5 > RPL18 > TER > Tmub1 > Zdhhc15 > SNX6 > ATPase > TFAM > EIF > TPI > α-TUB > COX7*. The stability ranking at different developmental stages associated with infection of PWN was as the following: *TER > RPL7 > RPS5 > Zdhhc15 > EF1-γ > RPL18 > Tmub1 > SNX6 > ATPase > TFAM > α-TUB > EIF > TPI > COX7*. The stability ranking at pupae treated with PWN was: *RPL7 > RPS5 > RPL18 > EF1-γ > TFAM > Tmub1 > TER > TPI > ATPase > SNX6 > EIF > Zdhhc15 > α-TUB > COX7*, and at adults treated with PWN was *RPS5 > SNX6 > RPL7 > EF1-γ > Zdhhc15 > TFAM > Tmub1 > TER > COX7 > ATPase > TPI > EIF > RPL18 > α-TUB*. The comprehensive analysis showed that *RPL7*, *EF1-γ*, and *RPS5* genes were the most stable reference genes combination for total samples. *RPL7*, *RPS5*, and *RPL18* were the most suitable reference genes in pupae treated with PWN. *RPS5*, *SNX6*, and *RPL7* were the most suitable reference genes in adult treated with PWN (Table 2). *TER, RPL7*, and *RPS5* were the optimal reference genes at different developmental stages associated with infection of PWN.

### Validation of the Selected Reference Genes

**FIGURE 5:**
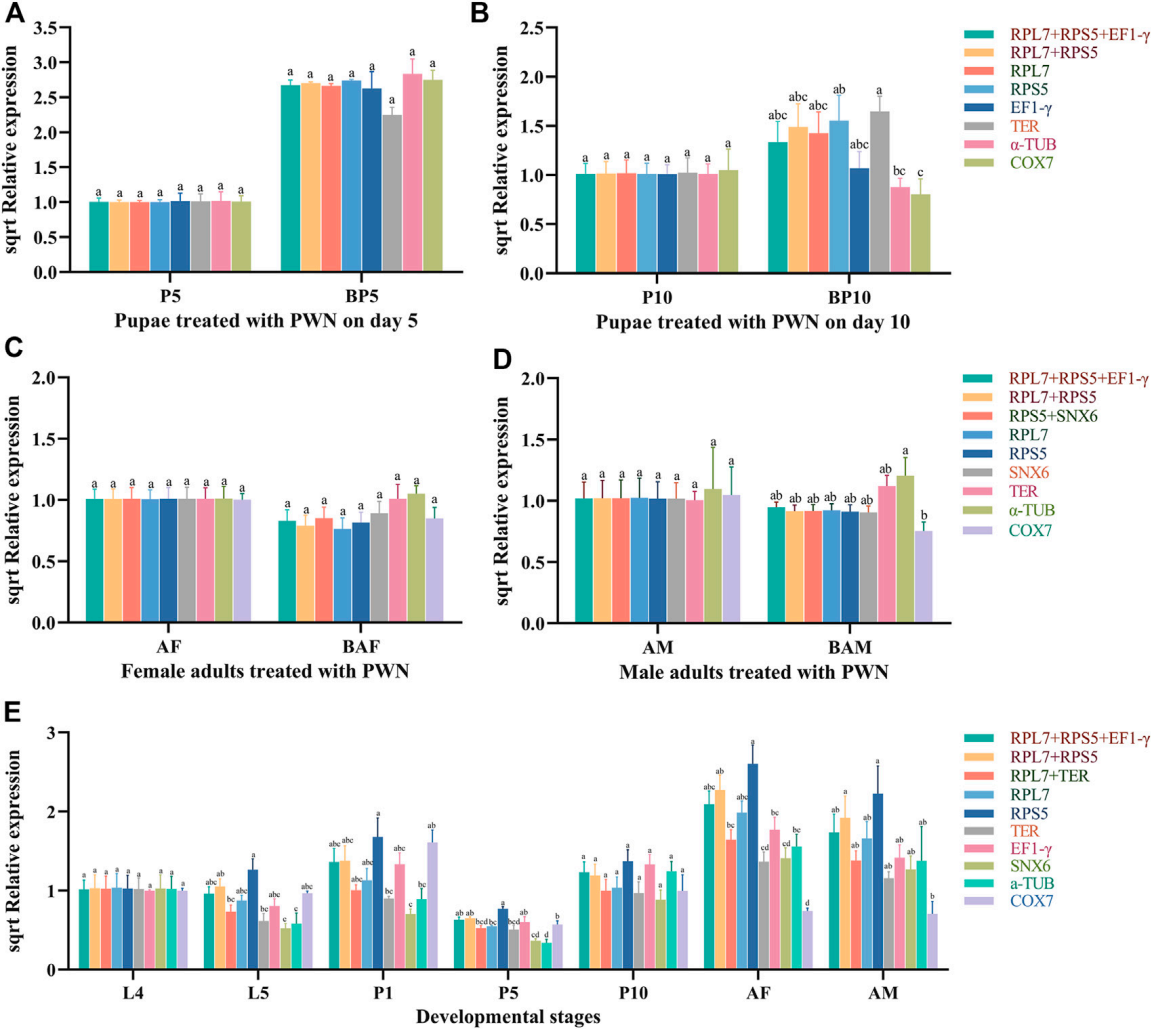
Relative expression levels of *KLF* normalized by candidate reference genes. Different letters indicate the significant differences in *KLF* expression levels (ANOVA, HSD, *p* < 0.05). Sqrt (Relative expression) represents the square root of the relative expression value. **(A)** Pupae treated with PWN on day 5. **(B)** Pupae treated with PWN on day 10. **(C)** Female adults treated with PWN. **(D)** Male adults treated with PWN. **(E)** Developmental stages associated with infection of PWN in *M. saltuarius*.

To verify the reliability of the selected reference genes, *KLF* was used as the target gene for RT-qPCR analysis. We used the four most stable candidate reference genes (*RPL7, RPS5, SNX6,* and *TER*), the combination of these stable genes (*RPL7 + RPS5 + EF1-γ, RPL7 + RPS5, RPS5+SNX6,* and *RPL7+TER*), and two most unstable reference genes (*α-TUB, COX7*) in different treatment conditions to normalize the expression of *KLF* (Figure 5).

In the pupae treated with PWN, the relative expression level of *KLF* was significantly up-regulated in BP5 and BP10 groups compared to control groups (P5, P10), which was normalized by the top-ranked gene (*RPL7*, *RPS5* or their combinations). Similar expression-profile changes were obtained by the combination of stable reference genes (*RPL7* + *RPS5* + *EF1-γ*), and there were no significant differences among those normalized by *RPL7*, *RPS5* individually, and *RPL7* + *RPS5*. However, the normalization by the least stable reference gene (*TER*, *α-TUB*, and *COX7*) led to a strong bias in the expression level of *KLF* in different treatments. *α-TUB* and *COX7* significantly decreased the transcription of *KLF* in BP10, and *TER* decreased in BP5. In the adults treated with PWN, although the expression trends were very similar, normalization with the unstable reference gene *TER* and *α-TUB* increased the expression level of *KLF* in BAF and BAM, which resulted in larger standard deviation values. At the different developmental stages associated with infection of PWN, the expression levels of *KLF* normalized by *RPL7* and *TER* individually, or *RPL7* + *TER* were different with *COX7* and *RPS5*. When normalized by *RPS5*, the expression of *KLF* increased in every developmental stage (L5, P1, P5, P10, AF and AM), and significantly decreased in AF and AM stages normalized by *COX7*.

## Discussion

*M. saltuarius* is a unique vector of *B. xylophilus* in northeast China. Its molecular physiology and the function of genes has been actively explored with the unpublished genomes, and recent transcriptomic advances have provided an opportunity for exploring the interspecific interaction mechanism between *M. saltuarius* and *B. xylophilus*, which was in favour of controlling the spread of pine wilt disease to north China. Therefore, it is necessary to probe gene function and quantify gene expression in *M. saltuarius*. Due to high sensitivity, rapidity, specificity, and accuracy, RT-qPCR is an effective method to study this mechanism. To reduce some inter-sample errors, appropriate reference genes are needed to normalize target genes (Zhao et al., 2022). However, there is no research on the reference genes of *M. saltuarius*. We systematically selected the reliable inference genes for standardization of gene expression by using five assessment algorithms (delta Ct, geNorm, NormFinder, BestKeeper, and RefFinder) in *M. saltuarius* at different developmental stages associated with infection of PWN and treated with PWN at the pupal and adult stages.

In our results, some candidate reference genes varied with different algorithms. *TER* ranked first in delta Ct and NormFinder, whereas it ranked sixth in geNorm and BestKeeper at different developmental stages associated with infection of PWN in *M. saltuarius*. The ranking of genes by different software was diverse, probably because different programs have different algorithmics, and the differences in the scaling systems used by the algorithms can also lead to these variations (Zhai et al., 2014; Sagri et al., 2017). Although the ranking order varies depending on the analysis program used, the overall trend was similar. For instance, in the adults treated with PWN, *RPS5, SNX6, RPL7, EF1-γ*, and *Zdhhc15* were all the top five stable genes in the delta Ct, geNorm, BestKeeper, and RefFinder. According to the geNorm, NormFinder, RefFinder, and BestKeeper, PWN, *RPL7*, *RPS5*, and *RPL18* were all the top three most stable genes in the pupae treated with PWN. Therefore, in practical application, the results provided by these algorithms are required to be considered comprehensively.

Most studies have found that two or more reference genes rather than a single reference gene can increase the accuracy of relative quantification (Vandesompele et al., 2002; Haller et al., 2004; Veazey and Golding, 2011). In our study, the optimal number of reference genes was calculated by geNorm. Most experimental conditions showed values below the proposed 0.15 cut-off value at V2/3. This result indicated that combining the top two reference genes would be adequate for the normalization of gene expression data at developmental stages associated with infection of PWN and PWN treatment conditions.

In this study, the stability of reference genes in *M. saltuarius* could differ under various experimental conditions. *TER* and *RPL7* were stable reference genes at different developmental stages associated with infection of PWN. At the same time, *RPL7* + *RPS5* and *RPS5* + *SNX6* were identified as optimal reference genes in pupal stage treated with PWN, adult stage treated with PWN, respectively. Previous studies have also shown that no reference gene has always been stably expressed under different experimental conditions, in which species, growth stage, tissue, temperature, strain, population, and pesticide varied. Sellamuthu et al. (2021) showed that *β-TUB, Eef2* and *RPS3* were the most stable gene under different developmental stages and sex, while *UBQ* and *V-ATPase* were the most stable genes after Juvenile Hormone III treatment in *Ips sexdentatus* (Sellamuthu et al., 2021). *β-TUB* expression was also stable in *Aquatica leii* at different developmental stages, but *GST* was the most stably gene under different temperatures (Yang et al., 2020). Similarly, *RPS32* was stably expressed in different tissues of *Agasicles hygrophila* while showing lower stability under different nutritional conditions (Guo et al., 2021).

Besides, it was observed that *B. xylophilus* induced more variations in the Ct values in *M. saltuarius*. In *M. alternatus*, PWN caused significant changes at the physiological and molecular level. Zhao et al. (2016) found that ascarosides secreted by dispersal juveniles (LIII) of *B. xylophilus* could facilitate *M. alternatus* pupation by upregulating ecdysone-dependent gene expression. When dispersal juveniles (LⅣ) of *B. xylophilus* entered the vector beetle, PWN affected the gene expression of Toll signal pathway (Zhou et al., 2018)*.* In this study, the stable reference genes at PWN treatment conditions were different from normal developmental stages. This result suggested that *B. xylophilus* can also cause variations in transcript levels in *M. saltuarius*.

Among the 14 reference genes studied in this study, ribosomal proteins exhibited more stability compared to other candidate genes in relation to different biotic (developmental stages and PWN treatment) factors. Ribosomal protein genes, which play an important role in ribosome biogenesis, protein translation, and cell development, were one of the most stable reference genes in diverse biotic and abiotic conditions in many insects (Zhou et al., 2015). In *Tribolium castaneum* and *Coccinella septempunctata,* ribosomal proteins exhibited a high level of stability at different developmental stages (Yang et al., 2016; Lü et al., 2018). In different sexes of *M ylabris cichorii,* and *I. sexdentatus*, *RPL22* and *RPS3*, respectively, were the most suitable reference genes for RT-qRCR normalization (Wang et al., 2014; Sellamuthu et al., 2021). Our results demonstrated that the ribosomal proteins were also transcriptionally conserved in *M. saltuarius* under PWN treatment.

The genes of tubulin, a protein that maintains the cytoskeletal structure and morphology in eukaryotic cells, are also frequently used as reference genes (Caridi et al., 2019). For example, *α-TUB* was stably expressed in *Drosophila melanogaster* exposed to different temperatures (Fleur et al., 2011). In *Antheraea pernyi*, *α-TUB* was suitable reference gene for normalizing RT-qPCR data infected by multicapsid nucleopolyhedrovirus (Zhao et al., 2019). However, *α-TUB* was unstable as reference genes under certain conditions, such as in *Spodoptera litura* larvae treated with azadirachtin (Lu et al., 2018). In our research, *α-TUB* showed instability under PWN treatment conditions. *COX* responds to a wide variety of metabolic states and is also considered a novel reference gene for different tissues in *A. hygrophila* and *Spodoptera frugiperda* (Guo et al., 2021; Shu et al., 2021), while was inconsistent with our results. In *M. saltuarius* subjected to several experimental conditions (different developmental stages, adults treated with PWN, and all samples), *COX7* was particularly unstable reference gene. These results suggested that reference genes differ from species to species.

*KLF* is a key DNA-binding transcriptional factor that regulates various pathways that pertain to insect metamorphosis, metabolism, and other cellular mechanisms, and was selected as the target gene (Weber et al., 2014). The overall transcription pattern of *KLF* normalized with the most stable internal reference genes was similar to the transcriptome data at different developmental stages associated with infection of PWN and PWN treatment. At different developmental stages, the expression level of *KLF* increased at emergence period (AF and AM) when normalized by the top ranked genes and their combinations. On the contrary, normalization with *COX7* showed the lowest transcription of *KLF* in adults (AF and AM). Under certain conditions, normalizing with unsuitable reference genes affected the gene expression and resulted in more significant standard deviations (Lü et al., 2018). The expression level of *KLF* in the pupae treated with PWN (BP5 and BP10) was higher than pupae treated without PWN when normalized by the top ranked genes and their combinations, and the same expression pattern was also observed in *M. alternatus* (Zhao et al., 2016). However, normalization by the least stable reference gene resulted in a strong bias. The transcriptions of *KLF* significantly decreased in the pupae treated with PWN (BP10). Similar results were observed in the condition of adults treated with PWN. Consequently, our findings confirmed the importance of selecting and validated accurate reference genes for RT-qPCR analysis to avoid the misinterpretation of target gene transcription data.

## Conclusion

This is the first study evaluating reference genes in *M. saltuarius*. We evaluated the stability of 14 candidate reference genes in samples from this beetle at different developmental stages associated with infection of PWN and PWN treatment conditions by delta Ct, geNorm, NormFinder, BestKeeper and RefFinder algorithms. We concluded that *RPL7* and *TER* were suitable reference genes at different developmental stages associated with infection of PWN. *RPL7* and *RPS5* were considered the most stable reference genes in pupae treated with PWN. *RPS5* and *SNX6* could be used as reference genes in adults treated with PWN. *RPL7*, *EF1-γ*, and *RPS5* could be used as stable reference genes in all the samples. Overall, *RPL7* and *RPS5* were the most stable reference genes for *M. saltuarius* under different conditions. Our results could provide stable reference genes for RT-qPCR gene expression analysis of *M. saltuarius*, also lay a foundation for the study of its phoretic relationship with *B. xylophilus*.

## Data Availability

The data presented in the study are deposited in the GenBank repository. The names of the repository and accession numbers can be found below: https://www.ncbi.nlm.nih.gov/genbank/; OM471799, OM471800, OM471801, OM471802, OM471803, OM471804, OM471805, OM471806, OM471807, OM471808, OM471809, OM471810, OM471811, OM471812, OM471813.
