# Supplementary material for: Identification and Validation of Reference Genes for Gene Expression Analysis in Monochamus saltuarius Under Bursaphelenchus xylophilus Treatment
Source: Front Physiol. 2022 Apr 25;13:882792. doi: 10.3389/fphys.2022.882792 (PMC9082747; doi:10.3389/fphys.2022.882792)
Supplement: Supplementary file 1 [file Table1.DOCX]

**Table S1**: List of 14 reference gene sequences used in the present study based on in-house transcriptome of *Monochamus saltuarius*.

>Gene_MSAL09320 sorting nexin-6 (*SNX6*)

ACGGATAAAACCTCTCCAGATGAGGGAAATAATGTTCCCCTGACGGATAGTTCTCTGGTGGTTGACATTTCAGATGCATTAAGTGAAAAAGATAAAAAACAGGAATTTTTGGTAGTAAGACAACATGAAGAATTTATTTGGCTTCATGATCGTTATGAGGAGGAACCCAAATATGCAGGGTACATTATACCACCTCCACCACCCCGTCCAAATTTTGATGCATCCCGTGAGAAATTGCAACGCCTTGGAGAAGGTGAAGGAACCATGAGGGAAGAGTTCAATAAAATGAAACAAGAGCTTGAGGCTGAATATTTAGCAACATTTAAGAAGACTGTAGCTATGCATGAAGTTTTTCTAACTCGATTGGCCAGCCACTCGATTTTTAGAGAAGATGCTCATCTGCACGTGTTCCTAGAATATGATCAGGACTTATGTGCGCGACCTAAAGGCAAACTCCAACAATTAGGCGGTTTAATTAAATCTGTCGCAAATGAGTTCCTTGACGGATTACCATGGTCATCTCAAGGAAGCCACATCTCGACAGACAAGATGACTGAGAAACATAAAGAACTTGCGGATAGTTATATAAAAATCTCTGGAAGTTTATTGCAATTGGCTAACAGTGATAATGGACATTTAGACAAATTTTTATCTAAAGTGGCAGACACATTTGAAAAGGCTAGAAAAATTGAAAGCAGAGTTGCTAGTGATCAGGACTTAAAACTAGCTGACACCCTTCGCTACTATATGAGAGACAGTCAAGCAGCACGAGCGTTACTCGTCAGACGCTTGAAATGTTTAGCAAACTATGAGAATGCCAACAGAGCCCTTGAGAAGGCCAGGCACAAAAACAAAGATATTCATGCGGCTGAAGCTGCCCAATCAGCTGCTTGTGAACAGTTCGAGGCAATTTCAACACAGGCGAAAGAAGAATTATTAGATTTTAAAACAAGACGTCTGCATGCATTTAGAAAAGGTAATGACTTATCATTAATTGCATAA

>Gene_MSAL04397 probable phospholipid-transporting ATPase (ATPase)

ATGGAATATCCTTTACAAGATTTTGACTGCGACAACGAAACAGAATTTTTACTTGGCCATTCGTCGAGTCATGGAAATGAGATGGATGTTCACACAGCTAGGACCAACCGCAACCCAAAAAGGCGTAGGAGCCTAGGAAGCATACTCTGCAGTTGTTGGTGTTCCATTTTTCGAAAATGTTGTGGTCCGAGGGAACTAAGATCTCGAAATATACTGCTGGGGAAACCGACCCCTGGTCAATTTCCAGCAAACGCTGTGAGAAACCAGAAGTATAACATAATAACCTTCTTGCCCTTGGTCCTCTTCCAACAGTTTAAATTTTTCTTGAATCTTTATTTCTTGATAATGGCAATTAGTCAATTCGTACCCGACATCCGTATTGGATATTTGTATACATATTGGGGACCTCTGTGTTTTGTCCTTCTGGTAACGATATCTAGAGAAGCAATAGACGACTTGAGGAGACATAAAAGGGATCAGGAAGTTAATAACCAGAAATGTAAAAGGTTACTGAACGATAGAAATAAACCGTTTGAAATTGTTGCTGCTCACAAATTAAAGGTTGGTGATTTGATTATTGTCGATAAAGATGAACGAGTACCTGCCGATTTAGTATTATTGAGAACTTCAGAAAGTAGCGGAGCCGTGTTTGTCAGAACCGACCAGTTGGACGGAGAGACTGACTGGAAACTTAAACTGGCCGTTCCGGCAACGCAAAAACTGTCCACGGACCATCAGCTGTTTGAAATCAGCGCCAGTATATACGCTGAGAAGCCCCAGAAGGACATTCATTCTTTTATAGGAACGTTTAGTCGGATGGACTCTGTAAATAATGACGAGAGTTTGGATTTGGACAACACTCTGTGGACTAATTGTGTTATTGCTTCCGGGCAAGCTACTGGGGTTGTTATTTATACAGGACCCGAAACCAGATCTGTGATGAATAATTCAGCGCCCAGATCGAAGGTGGGTCTGTTGGACATCGAAGTGAATACGATAACAAAGTTGTTGTTTGCCGCCGTGATTGGTCTGGCTTTACTGATGATGGCCTTGAAAGGATTCAGTGGACCTTGGTACCGTTACATGTTCAGATTTATTCTCTTATTCTCATATATTATTCCTATAAGTCTGAGGGTGAACTTGGACATGGGCAAGTCCTTCTACTCCTGGGCGATAGGAAAAGATCCGGCGATGAAGGGTACGACCGTGCGTTGCACGACCATCCCCGAGGAACTCGGCAGGATCTCTTATTTGCTCTCCGACAAAACGGGCACCCTCACGCAGAATTCCATGGTACTGAAAAGGCTGCATTTGGGTACGGTCAGCTATGCCGCCGACAGTTTCGACGAGCTAAGCATGGTCCTGAAGAGTTGTTTCACGTCTATGGAGAGTACCAGCGCGGGTGCTGCCAAATTCAAACGATCAGAAAACACGAGGATTAGGGATGCCGTGCAAGCGTTGGCCTTGTGCCATAATGTCACGCCGGTGTATGAGACTAGCGAAAATTGTGATACAGGGATTTCAGGTTCAATAACTTCAGAGACTGAGGCCGATCAGCACCTGCAGATGCCTGACAAGGCTGTGACGTACCAAGCTGCCAGTCCGGACGAGGTTGCCCTCGTCCAGTGGACGCAAGAGGTCGGTTTGACGCTCAGCAGGAGGGATCTTTCTTCCATGCAGCTGAGAGGACCCGACGGAAGACTTTTGAACTACGCCATTCTGCAGATATTCCCCTTCACCAGCGAGACTAAGAGGATGGGAATTATCGTTAAGGATTTGCACACGGGAGAGATCTTGTTTTATTTGAAAGGCGCCGATGTTGTTATGTCTGGTATCGTACAATATACAGATTGGTTGGACGAGGAGGTCGGAAATATGGCCAGGGATGGATTACGTACTTTAGTCGTGGCCAAGAAGGTCCTCACCGAAGAACAATATTTAGATTTTGAGACCCGGTACAACGTCGCACGTTTATCTACAACGGATAGAGTTGCGAGAGTAGCCCAGGTCGTGGAGTCCCTAGAAAGAGAAATGGAACTTCTGTGTATAACTGGCGTCGAGGACAAATTACAGGACAATGTGAGGGCGACTTTGGAGTTGTTGCGGAACGCCGGCATTAAAATTTGGATGTTGACCGGAGATAAGCTTGAAACGGCTACTTGTATCGCCAAAAGTTCCAGACTGGTATCCAGAACGCAAGGTCTTCACGTATTCAAGAAAGTCGTGACCCGCACAGACGCACACTTAGAACTGAACGCGTACAGGAGGAAACAAGACTGCGCCCTGGTGATAAGCGGCGAAAGCTTGGAGGTGTGTCTGTCGTACTACCAGCAGGAGTTCATGGAACTTGCTACTGCTGCTCCTGCCGTGGTTTGCTGCCGATGCTCTCCCACTCAAAAAGCGCAAGTGGTACAACTAATCCAAAAACACACGGGAAAGAGAACTGCAGCGGTGGGCGATGGCGGTAATGATGTCAGCATGATCCAACAAGCAGATGCCGGAATAGGTATCGAAGGACGCGAAGGCAAACAGGCGAGTTTGGCAGGGGATTTCAGTATCCCTCAGTTCTCGCAGTTGGCAAGGTTGTTGTTGGTGCACGGCAGGAAGTCTTATAAACGATCCGCTTCCCTGGCACAGTTTGTAATACACAGAGGTCTTATTATTTCTACTATGCAAGCGGTGTTTAGCAGCGTTTTTTATCTCAGCTCTGTCGCTTTGTATCAAGGATTCCTCATGGTTGGATACGCTACTGTATACACAATGTTTCCTGTTTTCAGCTTAGTACTGGACCAAGACGTCAGCCCCGAAATAGCGCTAACCTACCCCGAACTGTACAAAGAACTGGCTAAAGGAAGAAGCCTTTCCTTCAAAACCTTCTTCATGTGGGTATTGATTTCCATATACCAAGGTGGCGTGATAATGTACGGAGCTCTACTTTTATTCGAAGACGAATTCATCCACATCGTAGCCATCAGCTTCACTTCGCTGATCCTGACAGAGTTGATAATGGTCGCGTTGAACATCAGGACGTGGCACTATCTCATGGTGTTGGCGGAATTGTTCTCCTTAGGCCTGTACGGCTTGTCGCTGATAGTTCTACACGACTATTTCGACGCGGAGTTTATTAGAACAAAGGATTTCTTCTGGAAAACGTTAGTGATAACCCTGATATCTTGCTTGCCGCTTTACATCCTTAAATTCCTGAGGAAGAAGTTTTCGCCGCCCAGCTACAGCAAACTCTCTTAA

>Gene_MSAL02314 palmitoyltransferase ZDHHC15 isoform X2 (*Zdhhc15*)

ATGGCTGTACCTAGTGGTGATAATAGAGGACCATGCTACTGGTGTTTTAAGACTGTGAAATGGATACCAGTTTTGTTCATTGTAACTATAGTTGCATGGTCCTATTATGCATATGTTATTCAACTATGTTTATTGACTGTGGAAAGTGCGACAAAACAAGTTATTTATCTAATATTTTACCATGTCTTTTTCTTCATGTTTTGCTGGTCGTATTGGCAAACTATTTTTACCGATATTGGTAGAGTGCCTAGTAAATATAAAATTCCAGAAGCTGATTATGAAGCTTATTTGAACCACAGTGATTCTTATGAAACACAAAATACAATATTAGAGACATTTGCAAGCCATTTACCTATAACGAATATAACAATAAGTGGTGGTGTAAGATTTTGTGAAAAGTGTAAAGTTATAAAACCTGACAGAGCACACCATTGCTCGGTATGTGGCGTGTGTGTTTTAAAAATGGACCATCATTGCCCATGGATTAATAATTGTGTTTCATTTACAAATTACAAATTTTTTGTGCTTTTTTTGGGATATGCACTTACATATTGTGTATATGTATCATTAACTTCACTTCCATACTTCATTTCATTCTGGAGGGGTGATCTTCAAGGAATGGGAAGGTTCCATATTTTGTTTCTATTTTTTGTTGCAATAATGTTTGGTGTCAGTTTAATGTCCCTTTTCGGATATCATTGTTATTTAGTATGTGAAAATAGAACAACGTTAGGTCAGTATTTTTACAGAATGGAAGACATTTATAATAATGTGTATTTATATTTAATAGAAGCATTTCGACCACCAAGCTTCCGAGGTGTTGGTACAGACAAATACGGTTTTCACATAGGAAGGTATAAGAATTTTAAACAAGTGTTTGGAGATGACTCAAAAAGCTGGTTTCTTCCCATTAGTACTAGTTTGGGTGACGGTATAGAATACCCTGTACACTCACGCCACCAGCCGAGTTCTTATCATTCTATGGACAGCACCCAGAACAGCACTCGAGACGCTGAAACTGAAACAAGTAAGCTCAACCCTGGCCTTACAGAAATATCCGTATTGTAA

>Gene_MSAL10600 transcription factor A, mitochondrial-like (*TFAM*)

ATGGCAGGTCGAGTTTTATTTTTCAGGACTTGCAACCTTTTGTCAAATAGCCGATCCCTCTTAAACAATAGGATATTAAACGTTAATCTACTTCCGTCGCTAAGAGATTTAAAGCAAGAAACTCTTGAGAAATTGAAACTATTGAAAATTCCGGAGAAACCAAAGAGACCGCAATCTCCTTACGTTAAATTTGTTGCTGAACATCGCATTAATGTTATAAAAGATAACCCAAACTTCAAACAAACCGAAGTTATTAAAAAGTGCGCAGAGGATTGGAAAAGTATTACTCAAGAACTCAAAGAAGAATATAATGTTGCATATAAAAATGAATGTGCAATATATGATCAAAAGTTATCAACCTTTAATGCTAGTCTTACTCCAGAACAAAAAGAAGCTTTGCAACTTGCCAGTGAGGAGAAAAAATCAAGTAAAGAAAGGAGGAAAATCAAGAAGATTGAAAAGGAAACCAACAAACCTAAACGTCCAATGGGCGCTTACGCACTTTATATAAAGGAACAGAGCCAAATCAAAAATGTCTCTATGAAAGATCTGATATCAAGTTTAAAAGATGATTGGACTAAATTATCAGAAGACGAAAAGACTAAATACAAAAACCAATTTTTAAAAGAAAGAGAAAAGTATGAAACGGCAATGGCAGACTGGGAAGAAAAAATGTTAAAAGAAGGAAGAGATAATCTCATTAGGGTAAAATCTAAGGGCAAAAAAATAAAAACTTCCCGAATTAGACAGTTGAAACCAGGCAGTCCCCAATAG

>Gene_MSAL00760 60S ribosomal protein L18 (*RPL18*)

ATGGGTATCGATATTAATCACAAATACGACCGTAAGGTTAGACGTACAGAACCTAAAAGTCAAGATGTATACCTACGACTTCTTGTAAAGTTGTATCGCTACTTGGCTCGTCGTACAGACGCTAAATTCAACAAGATAATATTAAAGAGATTGTTTATGAGCAAGATATACAGGCCTCCAATTTCATTAGCTAGAGTTGTACGTTTGATGAGTAAACCTGGGCGTGAAGGACTAACAGCAGTCATTGTTGGGACAGTAACTGATGATTCTAGAATTTTTGAGGTTCCTAAATTGTCTGTTTGTGCACTACGTGTAACTGAAACTGCTCGCTCTAGAATCCTTAAAGCAGGTGGTGAGGTGATCACTTTCGATCAGTTGGCTTTGAGAGCCCCAACTGGTTCCAAAACGGTATTGATGCAAGGTAGACGCAATGCCCGTGAAGCTGCCAAGCACTTCGGACTAGCTCCTGGAGTGCCACACAGTCACACTAAGCCACTAGTACGTTCCAAAGGACGCAAATTTGAGAGAGCTCGTGGACGTAGGCGTTCGTGCGGATACAAAAAGTAA

>Gene_MSAL00096 60S ribosomal protein L7 (*RPL7*)

ATGGCACCAACAGCTGATAAAAAGACCAAAACAGTAAAGGGTCTGCCAGCAGTGCCCGAGTCTGTTTTGAAACATCGGAAAAGGCGTGAGGCTTCCAGAGCGAAACGTTTACAATCTGCGATCAAAAAGAAGTCTGAACAAGTCAAAAAACGTAAAGAAATTTTTAAACGTGCAGAGCAGTATGTAAAGGAATATAGATTGAAAGAACGCGATGAAATCAGACTTATTCGTCAAGCAAAAACTAAGGGCAACTTCTATGTTCCTGGAGAAGCAAAGTTGGCTTTTGTAATTCGTATTAAAGGTATAAACAAAGTTGCCCCAAAAGTACGTAAAGTTCTTCAGCTGTTCCGTTTACTTCAAATTAATAATGGAGTTTTTGTGAAATTGAACAAGGCTACCATTAATATGTTGAGAATATGTGAACCTTATATTACTTGGGGTTATCCTAACCTTAAATCTGTTAGAGAACTGATTTATAAGAGAGGTTTTGCAAAAATAAATGGGCAACGCATTCCCATAACTAGCAATCAGATTATTGAAGACAGATTAGGAAAATCTGGAATTATTTGTATTGAGGATTTAATTCATGAAATCTTCACAGTCGGTCCAAGGTTCAAATATGCTTCAAATTTCCTATGGCCCTTCAAGTTGAATACTCCAACCGGGGGATGGCGTAAGAAGACCAATCACTATGTTGAAGGTGGTGACTTTGGCAACAGGGAAGACAAGGTTAATGAGCTTCTCAGGAGAATGGTTTAA

>Gene_MSAL00148 40S ribosomal protein S5 (*RPS5*)

ATGGCTGAAGATTGGTCAGATGAAATACAGACGGCTGTTATAGCGCCGAAAATAGCAGAATTACCTGAAATCAAATTGTTCGGTAGATGGAACTGCGATGATGTGCAAGTATCCGATATGTCTCTTCAGGATTACATCGCAGTAAAAGAAAAAAATGCAAAATATTTGCCCCATTCTGCTGGACGATATGCAGCAAAAAGATTTCGTAAAGCCCAATGTCCCATCGTAGAGAGATTAACAAATTCCCTCATGATGCATGGGCGCAATAACGGTAAAAAACTGATGGCTGTTCGAATCGTTAAACATGCATTTGAAATAATTCATCTTCTTACTGGAGAAAATCCGCTACAGATTTTAGTATCAGCCATCATAAATTCTGGCCCCCGTGAAGACTCCACTCGTATTGGACGAGCTGGTACGGTAAGGAGACAGGCCGTTGATGTCTCACCATTGCGTAGGGTAAACCAGGCTATCTGGTTGCTCTGTACAGGAGCACGTGAAGCTGCATTCCGTAACATAAAAACAATCGCTGAATGTTTAGCCGACGAACTTATTAATGCTGCTAAGGGTTCCTCAAACTCATATGCGATTAAAAAGAAAGATGAACTTGAACGTGTTGCAAAATCCAACCGATAA

>Gene_MSAL03702 transitional endoplasmic reticulum ATPase TER94 (*TER*)

ATGGCTGATGGAAAAAGTCCCGATGATTTGGCCACCGCAATTCTCCGTAAGAAAGATCGTCCAAACAGACTTTTGGTTGAAGAAGCAGTCAACGATGACAATTCAGTCGTTGCTCTTTCACAAGCCAAGATGGATGAACTTCAGTTATTCCGTGGGGACACAGTATTGCTGAAGGGAAAAAGACGAAAGGAAACTGTCTGTATAGTACTTTCGGATGACACTTGTAGCGACGAAAAAATTCGCATGAACAGAAATGTGAGAAATAACCTGCGAGTACGTTTGTTAGACGTAGTGTCCATCCAGCCTTGTCCAGACGTAAAGTATGGTAAAAGGATCCATGTGCTTCCTATAGACGATACAGTAGAGGGTCTCACAGGAAACCTCTTCGAAGTGTACTTAAAACCATACTTCCTGGAAGCTTACCGGCCCATTCACAAAGACGATGTATTTGTTGTCCGTGGCGGCATGAGAGCCGTCGAATTTAAGGTTGTCGAGACAGACCCGGCACCATACTGCATCGTTGCCCCAGACACAGTCATTCATTGTGATGGCGACCCAATAAAACGCGAAGAAGAAGAAGAGGCATTGAATGCCGTTGGTTACGATGACATTGGTGGTTGCAGGAAGCAACTAGCGCAGATTAAGGAGATGGTTGAATTGCCTTTGCGGCATCCTTCTCTATTCAAAGCAATCGGTGTTAAACCGCCGCGCGGTATACTGCTGTATGGTCCACCAGGTACTGGTAAGACTCTTATAGCAAGGGCTGTTGCAAACGAAACAGGAGCTTTCTTCTTCTTGATCAACGGTCCTGAAATCATGAGCAAATTAGCTGGAGAGTCGGAGAGTAACTTGCGTAAGGCTTTTGAGGAAGCTGACAAAAATTCGCCGGCCATCATCTTCATTGATGAATTGGATGCTATTGCGCCTAAGAGGGAGAAAACTCACGGTGAAGTGGAAAGACGTATCGTGTCTCAACTTCTCACTTTGATGGATGGTATGAAGAAGAGCTCTCATGTCATCGTTATGGCAGCCACTAACAGACCTAACTCTATCGATCCCGCTCTGCGTCGCTTTGGTCGTTTTGACAGGGAAATTGACATCGGTATACCAGATGCCACCGGTCGTTTAGAAGTCTTACGCATTCACACTAAAAACATGAAGCTTGCTGATGACGTCGATTTGGAACAGATCGCAGCCGAAACTCACGGTCATGTTGGTGCTGATTTGGCCTCTCTGTGTTCCGAAGCTGCTTTACAGCAGATCCGTGAGAAGATGGACCTCATCGACTTGGAAGACGACCAGATCGATGCCGAAGTGTTGAATTCACTCGCAGTCACCATGGAGAATTTCCGCTACGCCATGACAAAGAGCAGCCCAAGCGCCCTCAGGGAAACTGTGGTGGAAGTTCCAAACATCACTTGGGACGACATCGGCGGTTTGCAGAATGTCAAGAAAGAACTGCAGGAACTGGTGCAGTATCCCGTGGAACATCCGGACAAGTTCCTCAAGTTTGGCATGCAACCGTCTCGCGGTGTGCTGTTTTACGGCCCGCCCGGTTGCGGTAAGACGCTTTTAGCTAAGGCTATTGCGAATGAATGCCAGGCCAATTTCATATCGGTAAAGGGCCCGGAGCTGCTCACCATGTGGTTTGGTGAATCCGAGGCCAATGTCAGGGACATATTTGATAAGGCCAGGTCAGCTGCTCCTTGTGTGCTATTCTTTGACGAATTGGATTCTATCGCAAAATCTAGAGGAGGCAACGTAGGAGACGCGGGCGGAGCCGCTGACAGAGTAATCAACCAAATTTTAACGGAAATGGACGGAATGGGTGCTAAGAAGAACGTATTTATCATCGGTGCCACTAACAGACCAGATATTATCGATCCGGCCATCCTGAGACCCGGAAGATTGGATCAGCTGATATATATCCCCCTGCCCGACGAAAAGTCCAGGGAGGCCATCTTCAGGGCGAATCTGAGAAAGTCCCCGGTAGCCAAGGACGTAGATCTAGTGTACATAGCCAAAGTGACGCACGGTTTCTCAGGCGCCGATCTCACGGAAATCTGCCAGCGCGCCTGTAAATTGGCCATCAGGCAGAGCATAGAGAGCGAGATCAGACGCGAAAGGGAGAGAGCTACGAACCCCAACGTTGCCATGGATTTGGATGAGGACGATCCGGTCCCGGAAATAACAAGGGCTCACTTCGAGGAAGCCATGCGTTTTGCCAGGAGATCCGTGTCGGATAACGATATCCGTAAGTACGAGATGTTTGCACAAACACTCCAGCAGAGCCGCGGGTTCGGAACGAACTTCCGTTTCCCGACGGCAGCGGGCGGCCCAGCCGCTACCGGAGGGACGGCAGGAGACCAGGCGAACTTCCAGGACGACCCCGAAGATGACTTGTACAGCTAG

>Gene_MSAL09667 transmembrane and ubiquitin-like domain-containing protein 1 (*Tmub1)*

ATGACACTAATCGAAGGTATAGGCGACGAGGTGACACATTTTTTTATCGCACTCTTCGCCGTAATTATAGTTACACTAGCTTGGTGGACCACTAATATATCTGAACAGAGGCATGTGAGGACAGTTTTGTTGCTAGAAAGGAGAAGGCACAGGGCTCACCGAAGACTGACCAATCATACAGAAACCGTCACTATCACGGAAGGATCTAGTATAGCACCTCCAATCACCGAAGAAAGTCCCATAGCTGTGTCAGCTACTACAGACTCTTCTATCGGTGACCAAATGTCTCACGAGGCTAGTACCAGTAATGAGGGTAGTACCATTACAGAACCTCCTGAAAATTTAAATCG

TGGGGAGGAGCAAAATATAATCGAAACAATGGATGCCGACGCTTGCGTTCTAAGGCAGAGGCGTTTGGCATTCTACGATAACTGGACTAGTGCAAATAGAGATGGTGGCGATGAATCCCGAGGTTCAGAAACCGCGGACCCTTCCCAAAGCCAAGACAGGATAGAAACGATACTAGACAGTAACGAACATACTGTAATGGAACATAATTATGCGGAACAGGACACATCGACTAGTTCGAATTGTAGGAACGAACAAAACTCGGAGAGTACTGAACGTAGTCTGCCTTCTGACAATAAATCTGGAATAACTATTAAATTAAAATATATCAATGATGATTTGAAGTTGGTTGATGGTAGGTTGGAGGAGATGTTGGGAGATTTTAAAAAGAGACATTTTCAAACAGAGTTATCTTCCAACAAGCTAGTCAGATTGATATTTAATGGAAGAGTGCTCCGCCCTGACACTCAAACCCTAAAGAGCTGTGGCCTCTTCGACAACTGCGTTGTTCACTGTTTGATCCACCAAAAAAGGGCACACGCGAACGAGAGTGGCGCCTCGGAAACAACAAGAGACGGTTATTCGTTTCCAGGCTCTGCCGGTATCAACAATATAAATAACAACAACCAAAACAGGGATTGGGATTTGGGGAATTTCTTGTTTGCATTTATCAGCTTTATTTTGCTAGCCGCGTGGTATTTTAGATACGTTTATGCTCACTTGTATACAGTTACAGCTACTGTAGGATTAATTTTGATCACGGGAATTTTTACGATAGTGTTGGTCGGAATGTATTTCCCAGACAACCCCGATCTACCCGCGCCACCCATCCGTTTTGCCCGAGAACGAGTGCAACCTCAACAGTAA

>Gene_MSAL09352 eukaryotic translation initiation factor 4B (*EIF*)

ATGGCTTCAGGTAAAAAGGGAAAGAAAACGAAGGGTAAAACCTTAGCCTTAACTGATTTTTTACAAGAAACCACTGGTTCTATACCCGCCCAACCTATACGCAAATCAAATATTAATTGGGCCGAAGAAGTGGAAGATTATGATGGCTATGACTCGAGGAAACCTATTAATGTTGTCCTTCCAACTGCTCCGAAGGCTTCAAGAGATTACGAAGATTTCAGTGATAAAGTGCCCAAAGAGCCGCCATACAGAGCTTATCTTTCAAATTTACCATATGATGTGGATGAGGAGGAAATTATGTTGTTTTTTAGGAATATGAGGATTGCAAGTATGAGGATTCCAAAAGATGACAGAGATAACACCAAACTGAAAGGTTTCGGATATGTTGAGTTTGAGGATAGAGACAGTCTGTTAAATGCTCTTGCCATTCCCGATACAACATTAAAAAACAGAAGGATCAGAATTGAAGTATCAGCAAACACAGATGACCGCAGAAGGGGGCGGATGGACATGGGTAGAGACAGGATGGATCGGGGCGAATCTACCGGTGATTGGCGTTCGGGGCCTCGTGTCGAGGTCTCTAATGGTGATCGTAGGGGAGGTTTCAACAGGGATCGTGATGGCGGTGGTTTTAATCGAGACAATATGAGAGGAGAAAGAGAAGAAAAGAGTTTCAGTAGAGACAATATGAGAGAAAGGGAAGAAAAGAGTTTCAGTAGAGACAATATGAGGGGAGAGAGAGAAGAGAGGAGTTTTAGCAGAGACAATATGAGGGGAAGCGACAGAGATGATAAAGGATTTAGAGATAATACTAGAGACGGAGGATTTAATAGAGATAGAGATAATACTCGAGACGGGGGATTTAATAGAGACAATAGAGATGGATTTAGACGAGATAGAGATGGTTTAGATGATAAACAAGGGGCATGGAGAGAGGGAGAAAGATTCAATAGAGATCAAGATAGAGGTTTTTCAAGAGACAGAGGTAATTATCGAGAATCAGATCGAATGAGCAGGTTCGACGATAGGGATGATCGTAAAGGATTCGGTAACAGAAGATTTGACAGTGACAGAGACAGGGATAGGAACGGTAGGGGCGGCGATAGATTCCAGGAAAGTGCTATGTCAGAACCAAGGGAAAGGCCAAAGTTGATGTTAGCACCTAGAACCAAAGCCGTGGAGAATATACCTGTGAAAGCAGAGTCAGTGCCTTCAGCTTCAATATTTGGAAGTGCCAAACCAGTAGACACTAGTCAGCGTGAACGGGAAATAGAAGAAAGGTTAGCAAAGGAACACTCGAACAAATCGAGAGATGCGAGTAGAGAGAGAAGTTCACACGTAGAAGAGAGCGATAAAAAGAATATAGAAAGAAGTAGGCCTCCAAGAAAAATAGAAGACAGCCCGAAAAGATATAATGATGAGAAAAAGGCGCCAAGAACTGAGTTCAAATCACCGGATAAAAATGAGAACAAACCTAAAATCGATAAGCCAAAGAAGGAGGAGAAAAGGGAAAAACATGAAAAAGAATTGCCTAAATTTACCGAGCCCGAACCACCAAATTTTGCGGCAAGCAACAAATTTGCGTTTCTGGAGACTGAGGATGCTTCGGATTAG

>Gene_MSAL06575 elongation factor 1-gamma (*EF1-γ*)

ATGGCTGCTGGAACTCTTTATACTTACCCTGACAATTTTCGTGCCGCAAAGACACTTATCGCGGCACAATATGCCAAAGCAAACGTCAAAGTAGCATCAAATTTCGTGTTTGGTGAGACAAATAAATCAAAGGATTTCCTAAAGAAGTTTCCAGGAGGAAAGGTGCCAGCTTTTGAGTCAAGCGATGGCAAATACCTGCAAGACAGCAACGCTATCGCTTATTTTGTGGCCAACGACCAGCTTAGAGGTAAAAACGAATACGATACTGCCCAAATATTGCAATGGATAGGATTTGCCGAAGGTGAAGTACTGCCAGCTGGTTGTGCCTGGGTTTTCCCAATTTTAGGCATTATAAGGAACAATGCTGGTGCCCAAGAAGCATTTCAGAGAGCGAAGGATGATATGAAGGCTGCACTAACAGTTCTCAACAGTCATTTACTTACACGCACTTATCTCGTTGGCGAACGCATTACCCTTGCCGATATTGTAGTGGCCTGCAATTTGCTTAATCCGTACAAGTATGTCCTGGATCCTGACTACAGACGGCCATTTGCTAATGTTAACAGATGGTTCACAACCTTAGTCAACCAACCACAATTCAAAGCAGTTCTAGGTGAAGTCGAACTTTGCAGTAAGGTTGCTCAGGCCGGATCTGTCGTTGCAGAAGGGAATTCTAAAAAAGTAGGTCAGGGAAAGCCTGGAAATGTCAAGGAACTTGGTTTTGAAAAATTGGAGAGAACCGTGAAGGAGGAGAAGAAACAAGAAAAGAAACAGCAACCCAAGAAAGAGACCCCCAAAAAAGAAAAAGAACCTGCTGAAGAATTGGATGCGGCTGATGCTGCTCTCGCCGCCGAACCCAAAAGCAAGGACCCGTTTGATTCCATGCCCAAGGGGACCTTCAACATGGATGATTTCAAGCGTTGCTACTCCAACGAGGACGAGAGCAAGTCGATTCCATATTTCTGGGAGAAATTCGATCCCGAAAACTATTCCATCTGGTACGGAGAGTACAAGTACCCAGAAGAACTGGCAAAAGTATTCATGAGCTGCAATTTAATCACCGGAATGTTCCAAAGACTGGATAAAATGCGTAAACAAGCTTTTGCCTCAGTGTGCCTTTTCGGAGAAGATAATAACAGTACCATTTCCGGCATCTGGGTGTGGAGAGGTCAAGAATTAGCTTTTCCTCTTAGTCCCGATTGGCAGATAGATTACGAATCGTACGATTGGAAAAAACTTGACGCCAAGTCGGAGGAGACTAAGAAGTTGGTCGAGCAGTACTTCTCCTGGTCGGGTACGGACAAGGGCGGGAGGAAGTTCAACCAAGGCAAAATCTTCAAATAA

>Gene_MSAL03188 cytochrome c oxidase subunit 7C (*COX7*)

ATGATCGGTAGAAGTAATCTGTTTCTTAGAAAAGCAGCTTGGAATTTGGCGAGGTACCACGATCATGGTGGTGTACCTGGAGCGAATTTACCTTTTTCCATTAACAACAGATACAAGCTAACAGCCTTCTTCATATTATTCTTTGGCTCTGGATTTTCAGCACCTTTCCTCATCTTGAGACATCAGTTACTTAAGAAGTAA

>Gene_MSAL09430 tubulin alpha-1 chain (*α-TUB*)

ATGCGTGAATGTATCTCAGTGCACGTCGGCCAAGCCGGAGTCCAAATCGGTAATGCCTGCTGGGAGTTGTACTGCCTGGAACATGGCATCCAACCCGACGGCCAGATGCCCTCCGACAAAACCGTAGGGGGTGGAGACGACAGTTTCAACACCTTCTTCAGCGAAACCGGGGCCGGCAAACACGTACCCAGGGCCGTATTCGTCGACTTGGAACCCACAGTAGTAGATGAGGTCAGGACGGGCACATACCGCCAGTTGTTCCACCCGGAACAACTCATCACAGGAAAAGAGGACGCTGCCAACAACTACGCTCGTGGTCACTACACGATCGGCAAAGAAATCGTTGACTTGGTCTTGGACCGCATCAGGAAATTGGCAGATCAATGCACGGGTCTACAAGGGTTCCTCATCTTCCACTCCTTCGGAGGTGGCACCGGATCTGGATTCACCAGTTTGTTGATGGAGAGGTTGTCTGTGGACTACGGAAAGAAGTCCAAGCTGGAGTTCGCCATCTACCCAGCTCCCCAGGTATCCACCGCCGTCGTTGAACCCTATAACTCTATTTTGACCACTCACACCACCCTGGAACACTCTGACTGCGCCTTTATGGTAGACAACGAAGCTATATATGACATCTGCAGACGTAACTTGGACATCGAGCGCCCCACATACACCAACTTGAACAGGCTGATCGGCCAGATCGTATCCTCCATCACCGCCTCTTTGAGGTTCGACGGCGCTCTCAATGTTGACTTGACAGAATTCCAAACTAACTTGGTCCCTTACCCACGTATTCACTTCCCTCTGGTCACTTACGCTCCCGTCATCTCCGCTGAAAAGGCCTACCATGAACAACTATCCGTGGCTGAAATTACCAACGCCTGCTTTGAACCCGCCAACCAAATGGTGAAATGTGACCCCCGTCACGGAAAATACATGGCCTGTTGCATGTTGTACCGAGGAGATGTCGTCCCTAAAGACGTAAACGCTGCTATTGCCACCATCAAGACCAAGCGTACCATCCAGTTCGTGGACTGGTGTCCCACTGGATTCAAAGTGGGTATCAACTACCAACCCCCAACTGTGGTGCCCGGAGGTGATTTGGCCAAGGTACAGCGTGCCGTGTGCATGTTGTCCAACACCACGGCCATCGCCGAGGCCTGGGCTCGTCTTGACCACAAATTCGATCTGATGTACGCCAAGCGTGCATTCGTCCACTGGTACGTGGGTGAGGGTATGGAGGAAGGAGAGTTCTCTGAGGCTCGTGAGGATCTGGCTGCTCTGGAGAAGGATTACGAAGAAGTAGGAATGGACTCCGGAGAAGGTGAAGGCGAGGGAGCCGAAGAGTATTAA

>Gene_MSAL06278 triosephosphate isomerase (*TPI*)

ATGAGTTTCCTCAGAGATATCAATAAACCCAGAAGGCTGCTGTTAAATCACTTGTCAGTTTTTCGACAAGTGAAAATTAAGATGGCCGAAACTGGGGAAACTGTAGAAGAAGCCCAAGGGGAGACTGAGAACACTGAGGAGCCTGTTGAGACAGTCGAAGCAATACAAGACCTTGTAGAAGAAGATAAAACAGGAGAAGCTGCACCGGAAGAAGCTAAGCCTGAAGAAACCACACCTGAAGAAGCGCCACCCGAAGAAAGTAAACCTGAAGAAGCTCCTGCTGAAGGTGAATCTCCACCAGAAGAAGGTAAAAGTGAGGAAGCTAAACCCGAAGAAGTTAGTAAACCCGCGGAACCAACTAAACCTGAAGAAGCAGAAGTCAAAAAAGAACCACCATGTACCGCTCTGTGTGCTACTATCCTCGACGGCAAGCCCGAAGGCGGAAAAAAACCTGTAGAAATGGCACGCAAATTCGTCGTTGGTGGCAATTGGAAGATGAACGGAAATAAGGAACAAATCGGCCAGATACTGAACTTTTTAAAAGAGGGGCCTTTGAATGCCGACACAGAGGTGGTCGTTGGCGTCCCTGCTATCTATTTAGAGTTTGTTAAAAATAATGCACCTGGAAACGTCGAAGTCGCAGCTCAAAATTGTTATAAGGCTGATAAAGGCGCTTTCACAGGTGAAATATCCCCACTCATGCTTAAAGATATTGGCGTTAACTGGGTCATTCTCGGTCATTCTGAACGTCGTCAGATCTTTGGCGAATCAGATGAGCTGATCGCAGATAAAGTAGCATTTGCCTTGCAAAACGGTCTGAAAGTGATCGCTTGCATCGGTGAGACCTTAGAGGAACGCGAGGCTAGTAAAACCGAGGAAGTCGTTTTCAGACAAACGAAAGCCATCGCCGACAAAGTCAAAGACTGGTCGAACGTGGTTATCGCTTACGAACCAGTCTGGGCTATCGGAACGGGCAAAACCGCATCCCCGGAACAGGCGCAGGAGGTCCATAAGGCTTTGAGAGATTACATTGCTAGTTCCATCAGCCCGGACGTTGCCGGTGGTATCAGGATCCAGTACGGTGGGTCTGTTACTGGGGCAAACTGTAAGGAGTTGGGATCGCAGCCCGACATCGACGGTTTCCTAGTTGGGGGAGCTTCTTTGAAGCCGGAATTTGTCGACATCGTAAACGCAAAACAGTGA

>Gene_MSAL01719 Krüppel-like factor luna (*KLF*)

ATGGAATGGTATTTGAAAGATGAACCAAAAATGAAAACTTACAAGACGCTACCTTCCGATATAAGTAGAATTTGGTATAAAGTAAATTGTATAGAAGATTTTAGTGATAGTCATTTTGATACATTGAGCTCAGCAAGTTCAACATGTTCATGGGATGGTGCTTTATCTTGTGCGGTATTAGTGAAACAGGAACCTGTAGATGATGAGGATGACGATGATGAAAATGAGGATGAAGAGGATAGTGATGATGAGGATCATGAGAGTTATGAAAATAGAGTACATTCTGATGTAAGAGATGCTTTACAATTGCGGTTAATTTCAAGAAATGCAGAGACTTTGACTCCTCCCTCTAGCCCTGAATCAGAATTAGGGCATAGTAATGGAAGTTCTGCTGCTAGTGATTTAGATGTTTGTGGGCTTCGCATTAGTAATACACAAGGACACCGAAATACAATAGTCAGAGTGCGAGCCTCTGGAGTTACTAGGTACATATCTGTAGTTCCTAGACCTCCAAGTCACATTTCATCATCATCAACAAACTCAACACCTACAAAACATCATAACCGTTTAGATCATTCACCAGATTCTAAAAGAAGAATACATAAGTGCCAATTTCTTGGATGTAAAAAAGTTTATACTAAAAGTTCCCATTTAA
