# Supplementary material for: Identification and Validation of Reference Genes for Gene Expression Analysis in Monochamus saltuarius Under Bursaphelenchus xylophilus Treatment
Source: Front Physiol. 2022 Apr 25;13:882792. doi: 10.3389/fphys.2022.882792 (PMC9082747; doi:10.3389/fphys.2022.882792)
Supplement: Supplementary file 2 [file DataSheet1.docx]

**Supplementary Material**


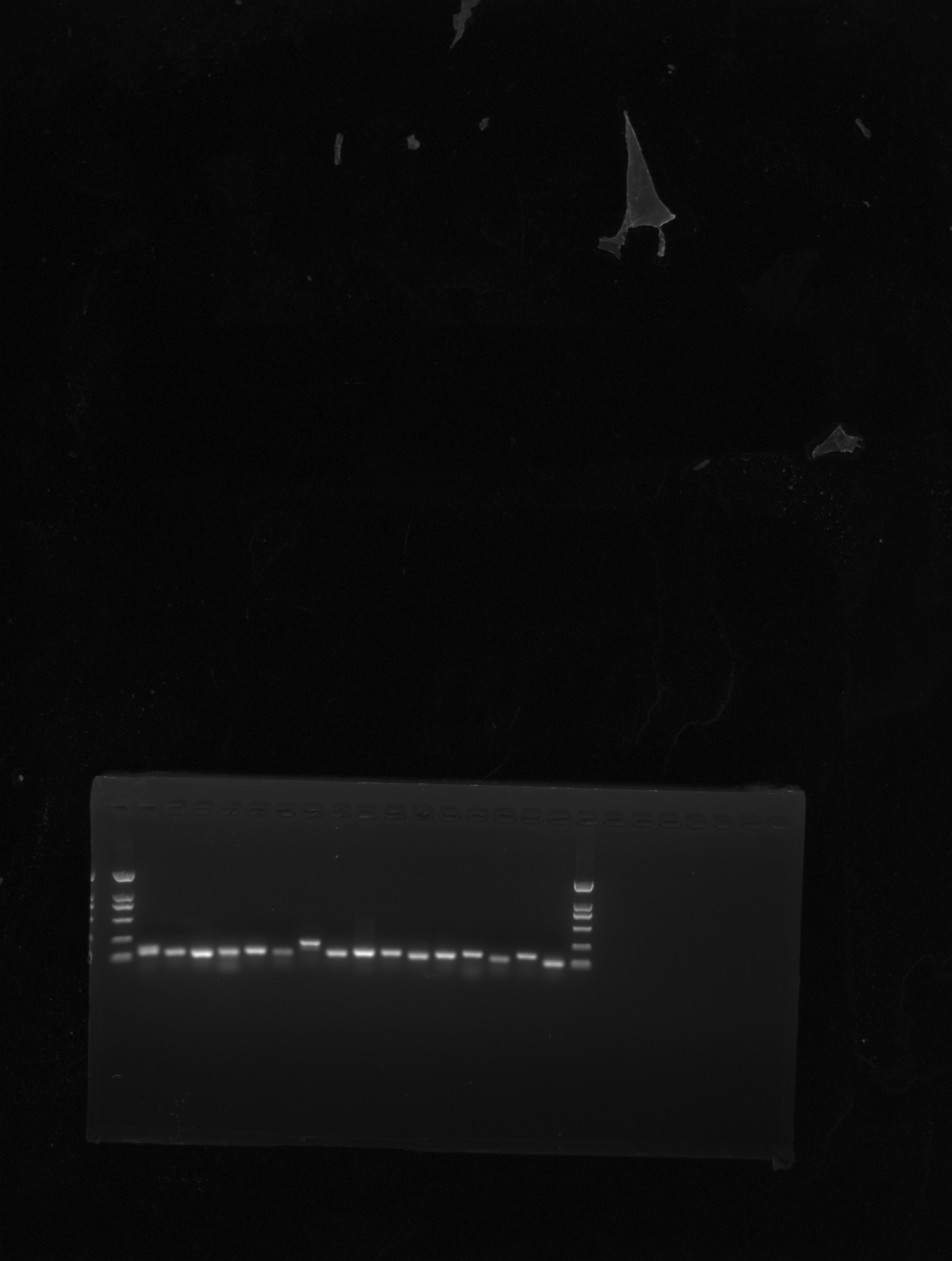


**bp**

**250**

***EIF***

**Marker**

***EF1-γ***

***TPI***

***α-TUB***

***COX7***

***Tmub1***

***TER***

***RPL7***

***RPS5***

**100**

***SNX6***

***TFAM***

***ZDhhc15***

***RPL18***

***ATPase***

***KLF***

**Figure S1** Amplified fragments of 14 candidate reference genes exhibited by agarose gel.


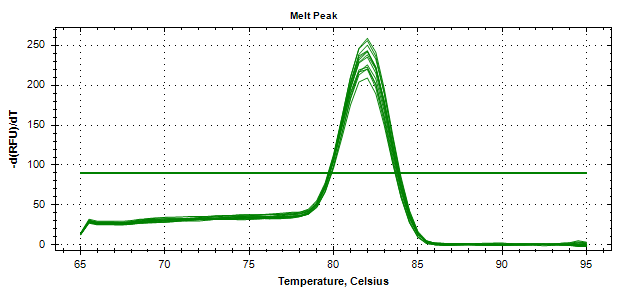


***α-TUB***


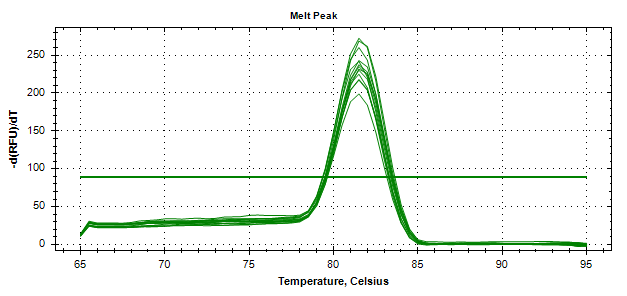


***EIF***


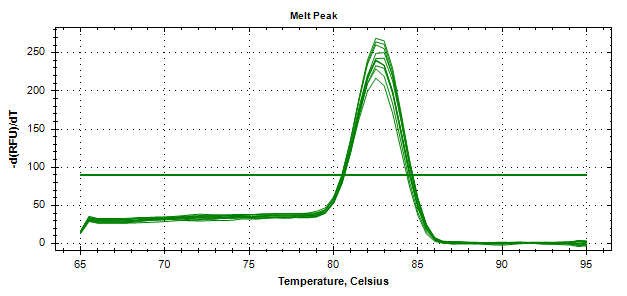


***TPI***


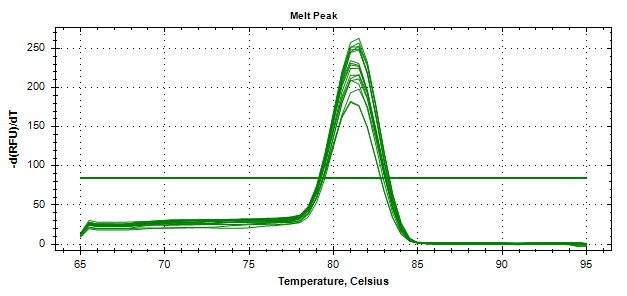


***RPS5***


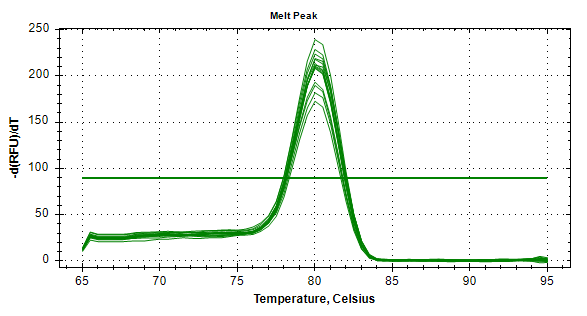


***EF1-γ***


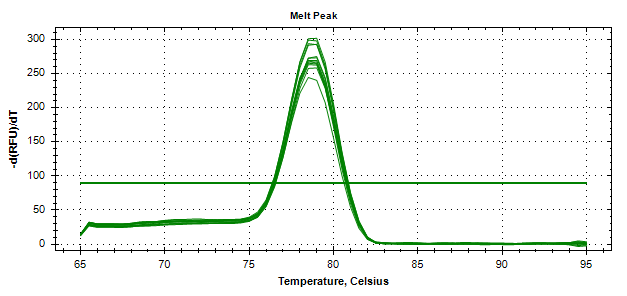


***COX7***


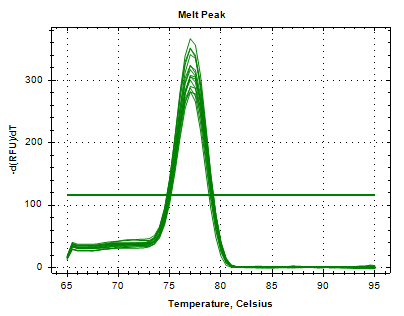


***RPL7***


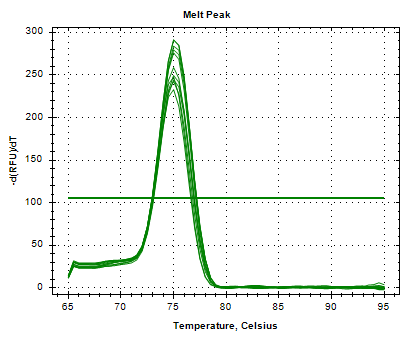


***Tmub1***


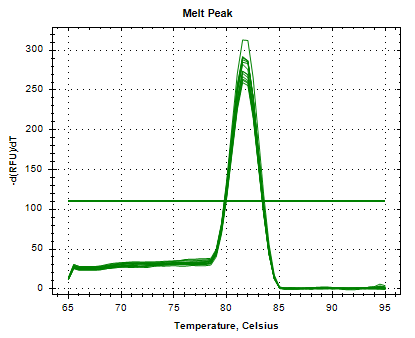


***TER***


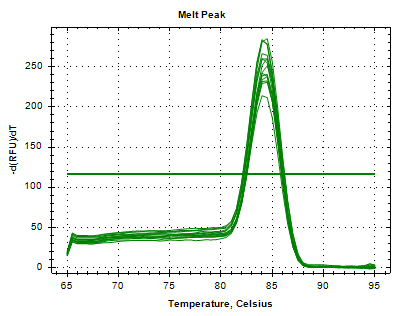


***RPL18***


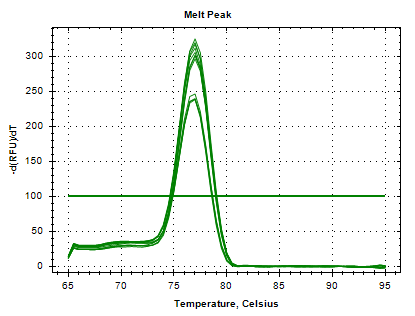

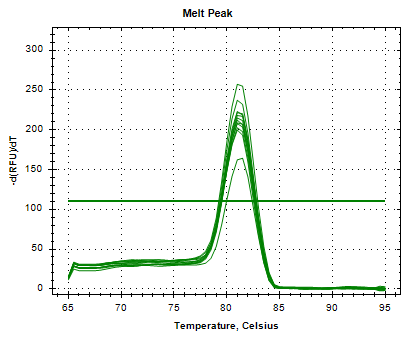


***TFAM***

***SNX6***

**
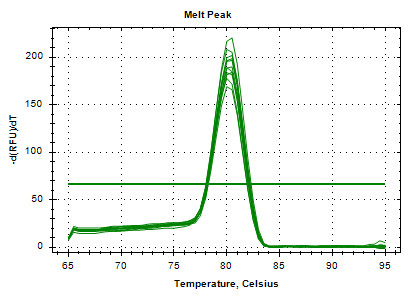

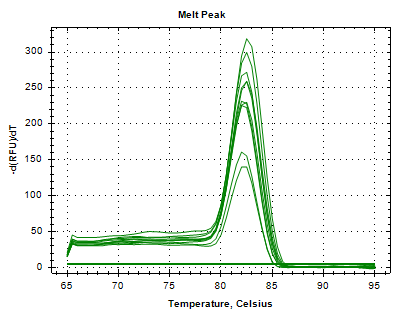

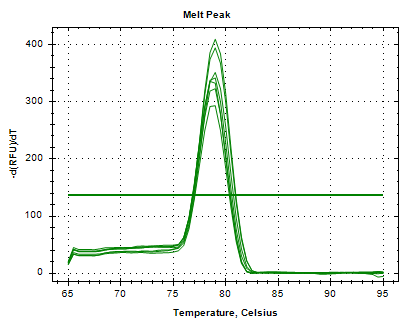
**

***ATPase***

***KLF***

***ZDhhc15***

**Figure S2** Melting curves for the candidate reference genes.


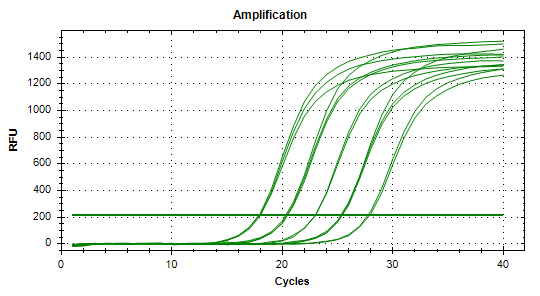

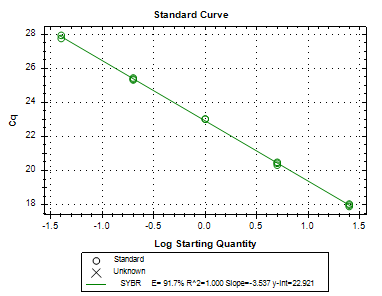

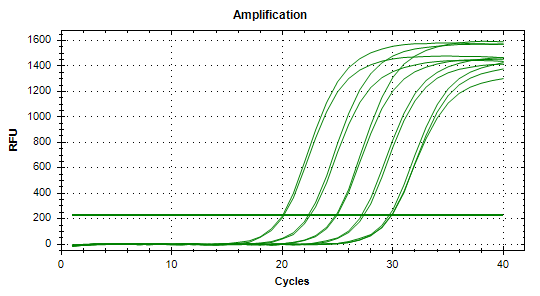

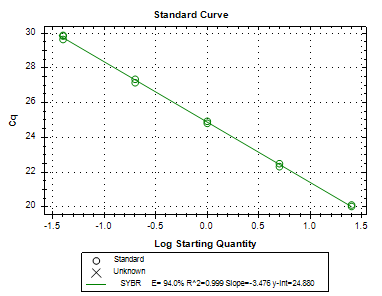


***TPI***

***α-TUB***


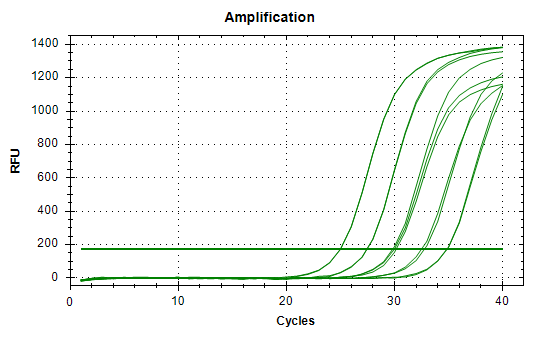

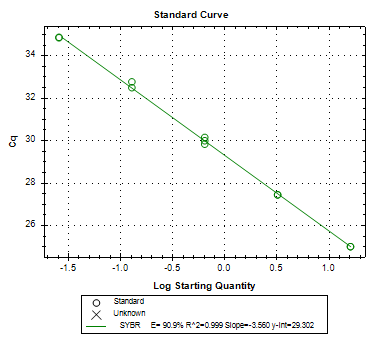

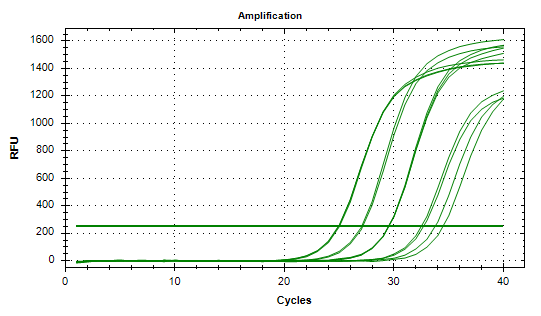

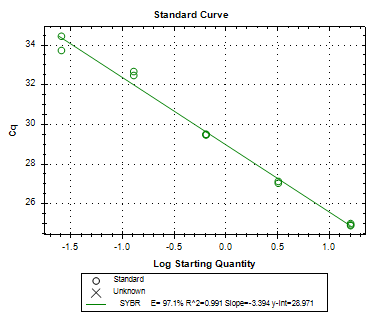


***TFAM***

***Tmub1***


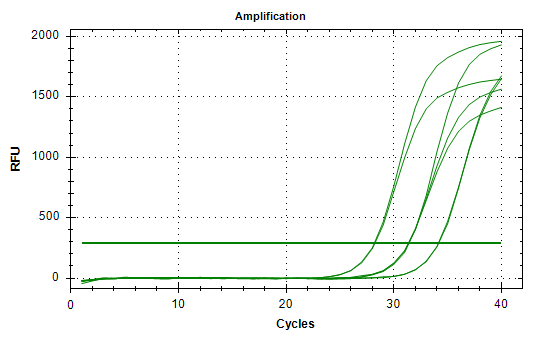

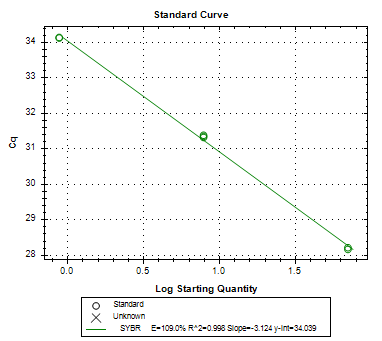

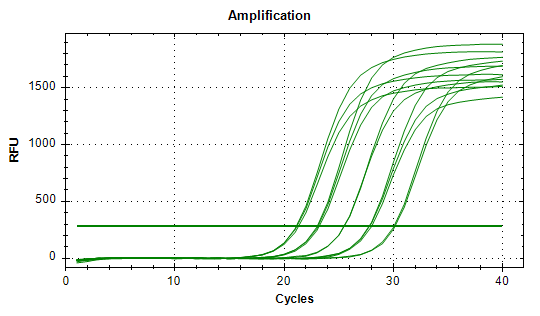

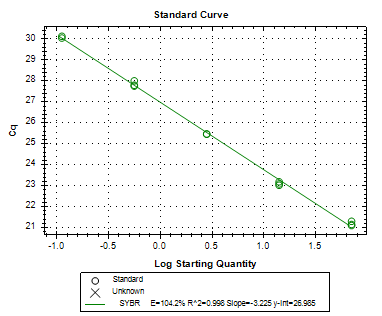


***RPL18***

***ZDhhc15***


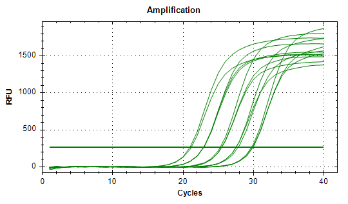

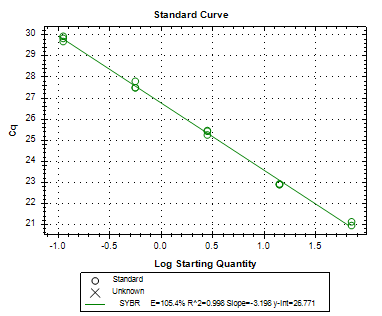

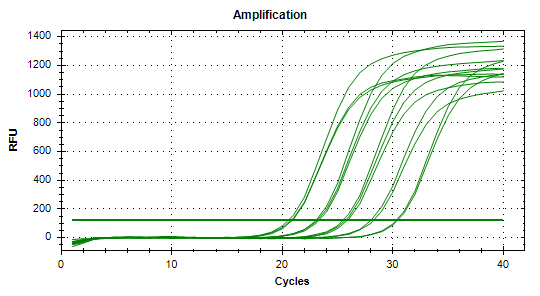

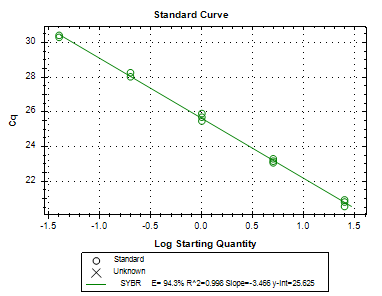


***EIF***

***RPL7***


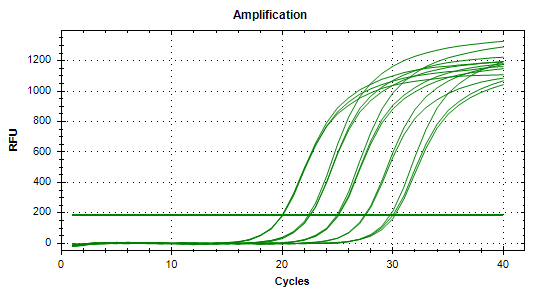

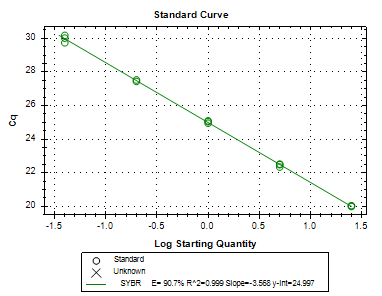

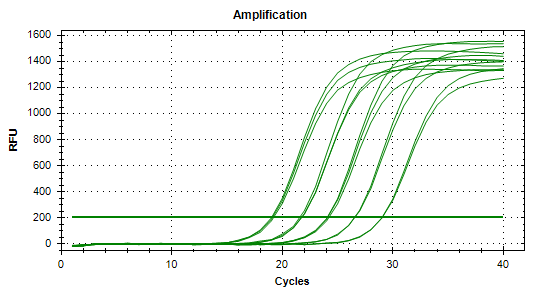

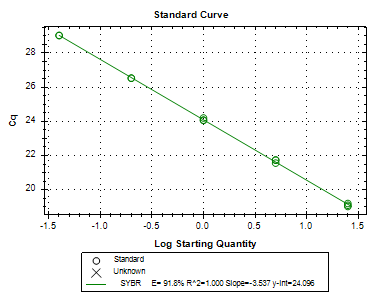


***COX7***

***EF1-γ***


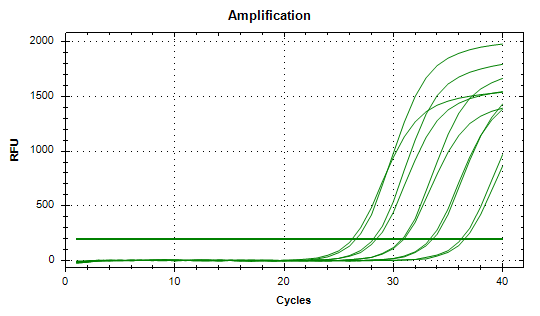

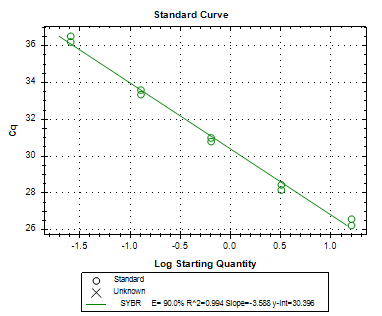

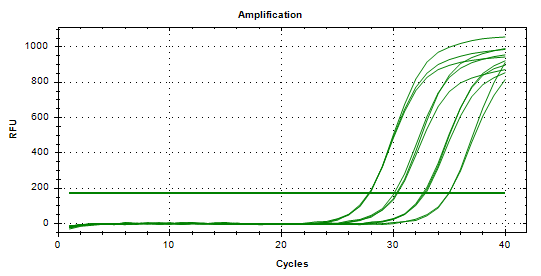

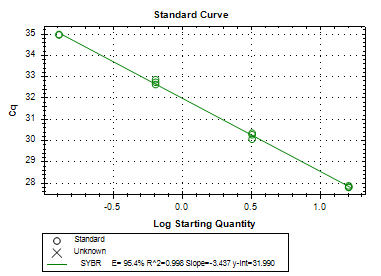


***KLF***

***ATPase***

**Figure S3** Amplifications curves and standard curves for the candidate reference genes.
